# Substituted 3-Benzylcoumarins as Allosteric MEK1 Inhibitors: Design, Synthesis and Biological Evaluation as Antiviral Agents

**DOI:** 10.3390/molecules18056057

**Published:** 2013-05-21

**Authors:** Chao Wang, Hao Zhang, Fengrong Xu, Yan Niu, Yun Wu, Xin Wang, Yihong Peng, Jing Sun, Lei Liang, Ping Xu

**Affiliations:** 1 State Key Laboratory of Natural and Biomimetic Drugs, Department of Medicinal Chemistry, School of Pharmaceutical Science, Peking University Health Science Center, Beijing 100191, China; 2 Department of Microbiology, School of Basic Medical Sciences, Peking University, Beijing 100191, China

**Keywords:** MEK1 inhibitor, coumarin, antiviral

## Abstract

In order to find novel antiviral agents, a series of allosteric MEK1 inhibitors were designed and synthesized. Based on docking results, multiple optimizations were made on the coumarin scaffold. Some of the derivatives showed excellent MEK1 binding affinity in the appropriate enzymatic assays and displayed obvious inhibitory effects on the ERK pathway in a cellular assay. These compounds also significantly inhibited virus (EV71) replication in HEK293 and RD cells. Several compounds showed potential as agents for the treatment of viral infective diseases, with the most potent compound **18** showing an IC_50_ value of 54.57 nM in the MEK1 binding assay.

## 1. Introduction

According to WHO statistics, almost 75% of infectious diseases are caused by viruses, yet R&D efforts related to the discovery and development of novel and potent antiviral agents have obviously lagged behind. A major difficulty in developing antiviral agents is drug resistance caused by virus variation. To the best of our knowledge, most studies of viruses and viral diseases focused on “viruses” themselves and ignored the interactions between viruses and their host cells. Several studies have reported that the RAF/MEK/ERK pathway plays a very important role in virus infection and replication. Selective blocking of the pathway can prevent the virus proliferation [1,2,3,4].

The RAF/MEK/ERK pathway belongs to mitogen-activated protein kinase (MAPK) pathways which are key intracellular signaling pathways involved in the regulation of a wide range of cellular events, including cell proliferation, differentiation, survival, apoptosis, *etc.* Functionally, the RAF/MEK/ERK pathway of normal cells is in a resting state, and this low activity state is sufficient to maintain the basic requirements of normal cell metabolism. As viruses need to activate ERK pathway continually when they replicate in cells [3,5], blocking of the pathway has a strong inhibitory effect on viral replication. Since the ERK pathway is coded by host genes, antiviral drugs targeting the ERK pathway can significantly overcome the drug resistance problems caused by virus variation.

MEK1 and MEK2 are closely related, dual-specificity tyrosine/threonine protein kinases that have an integral role in the ERK signaling pathway by phosphorylating the downstream ERK1 and ERK2. Since ERK1 and ERK2 are known to be the only substrates for MEK1 and MEK2 [6,7], targeting these two receptors had been an attractive approach for new therapy development. Lots of MEK1/2 inhibitors have been reported in the last twenty years, and Hasemann *et al.* have reported the crystal structure of ternary complex of MEK1 bound to its biarylamine inhibitor PD318088 and MgATP [8]. Most of the efficient MEK1/2 inhibitors are designed as non-ATP-competitive allosteric inhibitors [9,10,11,12]. They bind in a unique inhibitor-binding pocket adjacent to the ATP binding site, inducing conformational changes in the unphosphorylated MEK1/2 enzymes that lock them into a closed but catalytically inactive species [8]. This unique binding mode offers the non-ATP-competitive allosteric inhibitors better specificity and selectivity compared to the ATP-competitive inhibitors, which share a common ATP binding pocket and negatively influenced by the inhibition of other kinases.

Till now, most reported allosteric MEK1/2 inhibitors bear a biarylamine scaffold [6,13] and there are 13 MEK inhibitors at different stages of clinical evaluations [14] although none of them has been approved yet for clinical use. On the other hand, very limited types of non-biarylamines have been identified as MEK1/2 inhibitors, such as PD98059, U0126 and G8935 [15]. PD98059 was the first synthetic MEK inhibitor which only had activities *in vitro* [16]. Similarly, U0126, the second MEK inhibitor with better potency, was mostly used *in vitro* at research labs due to its serious toxicity issues [10]. The coumarin derivative G8935 was also identified as MEK inhibitor by TR-FRET-based *in vitro* assay, however, no more functional evaluations have been reported [15].

For years, we have been focusing on the discovery and development of novel MEK1/2 inhibitors, the evaluation of their biological activities and the mechanisms of their usage as antivirus agents. Our early studies showed that in cell-based assays replication of enterovirus EV71, borna virus and herpes simplex virus HSV2 could be effectively suppressed by the MEK1/2 inhibitor U0126 [17,18,19,20,21,22]. Selective blocking of mRNA expression of MEK1 could significantly inhibit virus replication, by contrast, knockdown of MEK2 expression showed dispensable effect, suggesting distinct functions of MEK1 and MEK2 in virus replication [22,23]. MEK1 might be a potential broad antiviral molecular target. Herein, we report the discovery of a series of novel 3-benzylcoumarins as allosteric MEK1 inhibitors. Multiple *in vitro* biological evaluations, including binding affinity to phosphorylated MEK1, ERK pathway inhibition and antiviral effects were performed, which demonstrated that these compounds were active MEK1 inhibitors and potential antiviral agent candidates.

## 2. Results and Discussion

### 2.1. Molecular Design

**Figure 1:**
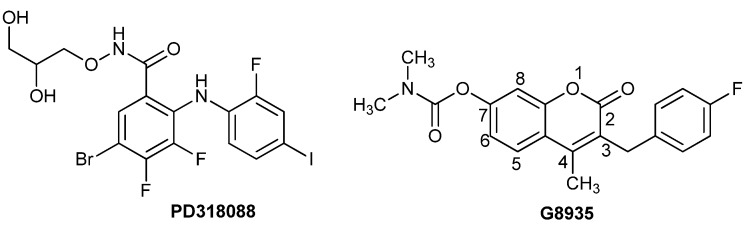
Structures of PD318088 and G8935.

According to the report by Gu *et al*. [23], a series of coumarin derivatives could potently inhibit the activation of the unphosphorylated human MEK1. Molecular modeling studies suggested that the best inhibitor, G8935 (Figure 1) with an IC_50_ of 0.3 μM, bound to the allosteric site in the unphosphorylated conformation of MEK1. We used G8935 as a lead compound and docked G8935 into the allosteric site of the MEK1 structure (PDB code: 1S9J) using the docking program GOLD [23] and compared its binding mode with the host biarylamine allosteric inhibitor PD318088 (Figure 1). 

**Figure 2:**
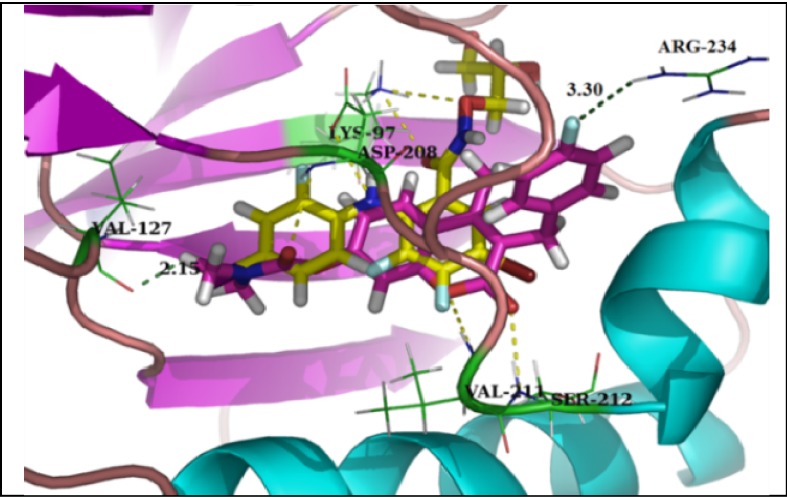
Superposition of the docked conformation of PD318088 (yellow) and the active conformation of G8935 (pink) (PDB code: 1S9J). Yellow dotted lines: hydrogen bonds between compounds and MEK1; green dotted lines: illustrative distance between compounds and MEK1.

**Figure 3:**
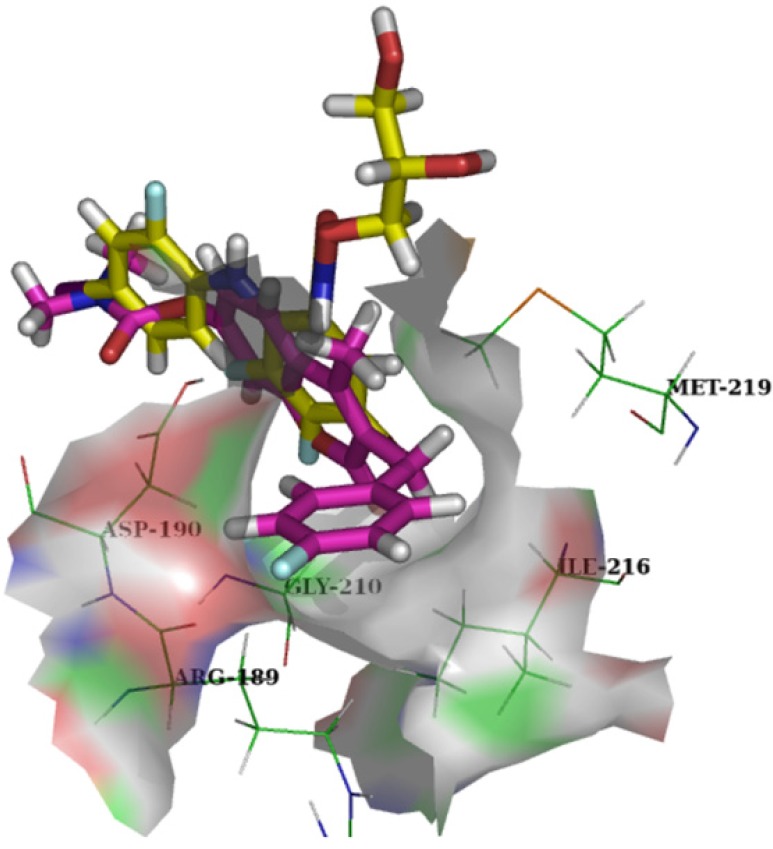
Close-up view of the 3-benzyl group fitting into the lipophilic pocket of MEK1 unoccupied by PD318088. Pink: G8935; Yellow: PD318088.

Their docking energy scores were similar to each other and the docked G8935 (docking score: 67.14) overlapped very well with the active conformation of PD318088 (docking score: 66.01) (Figure 2). Compared with the biarylamine skeleton, the 3-benzyl group is positioned in a lipophilic pocket formed by Met219, Ile216, Gly210, Arg189, and Asp190 that is not occupied by PD318088 (Figure 3) and the distance between the fluorine at the *para*-position of the 3-benzyl group and the terminal hydrogen of Arg234 is 3.30 Å, therefore structure modification at C3 to improve the affinity could be a very promising approach. The oxygen of the coumarin ring formed strong hydrogen bonds with Val 211 and Ser212, and the methyl group at C4 extended in the same direction as the side chain on the aromatic ring of PD318088, so optimization can be performed at the C4 position to enhance protein-inhibitor interactions. Moreover, the carbonyl group of the carbamoyloxyl moiety substituted at the C7 position also formed a strong hydrogen bond with the backbone of Asp208 in the DFG motif and the distance between the hydrogen of the methyl group in the C7 substituent and the carbonyl oxygen of Val127 is only 2.15 Å, so optimization at the C7 position can also be performed to enhance the interaction between the inhibitor and these two residues. 

**Figure 4:**
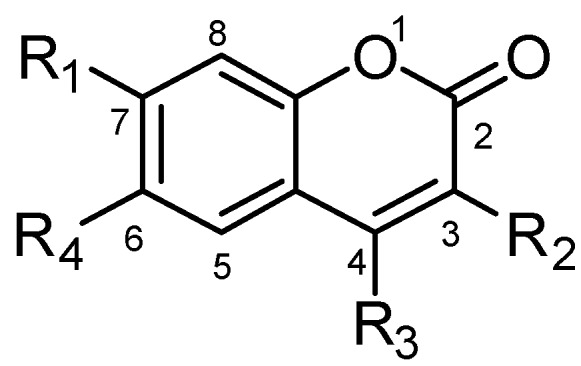
Structure and substituent groups of designed coumarin derivatives.

Based on the above observations, 3-benzyl- coumarin may represent a better scaffold for novel MEK1 inhibitor design compared with the biarylamines. As most reported allosteric MEK1/2 inhibitors bear a biarylamine scaffold and coumarin is one of the very limited types of non-biarylamine scaffold without further functional evaluations, we selected coumarin as the key moiety and performed structure optimization at the 3, 4, 7-postions to break though the biarylamine limitation and also evaluated biological functions of these compounds. This scaffold has following advantages: (1) the docking results suggested that the coumarin derivative overlapped very well with the biarylamine, suggesting a high possibility of similar MEK-inhibitory potency; (2) coumarin derivatives can be easily synthesized; (3) lots of studies could be done for coumarin-based MEK inhibitors since only one report [23] about coumarin structural modifications has been published and the highest potency could still be improved. Besides benzyl, a derivative with a benzoyl at the C3 position was also designed for comparison. Finally, a series of coumarin derivatives with benzyls or benzoyls at C3, various alkyl, cyano or carboxyl groups at C4 and different types of ester group at the C7 position were designed and synthesized (Figure 4). 

**R_1_** = OH; OCH_3_; CH_3_COO; EtCOO; (Me)_2_NCOO; (Et)_2_NCOO; *n*-PrCOO; BuCOO; *t*-BuCOO; PhCOO; PhSO_2_COO; *p*-CH_3_PhCOO; **R_2_** = Bn; 4-F-Bn; bezoyl; **R_3_** = H; Me; Et; *n*-Pr; CH_2_Cl; CH_2_OH; CN; COOH; **R_4_** = H; Cl.

### 2.2. Chemistry

**Scheme 1:**
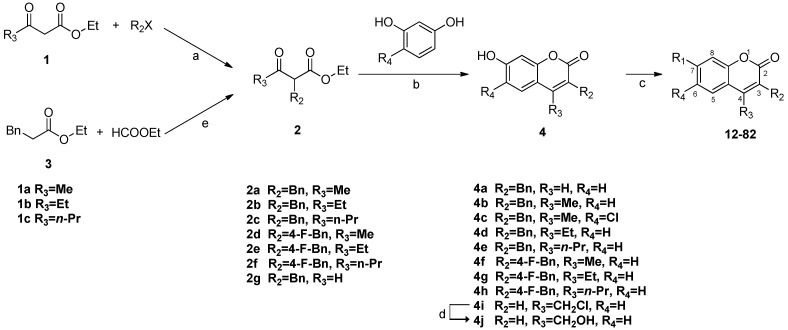
Synthesis of substituted 3-benzylcoumarin derivatives ^1^^,^^2^.

^1^*Reagents and Conditions*: (a): NaH, DMF, 60 °C; (b): 70% H_2_SO_4_, r.t.; (c): acyl chloride, NaH, r.t.; (d): H_2_O, reflux; (e): NaH, THF, 63.3%. ^2^ For R_1_, R_2_, R_3_ and R_4_ of target product, see Table 1.

**Scheme 2:**
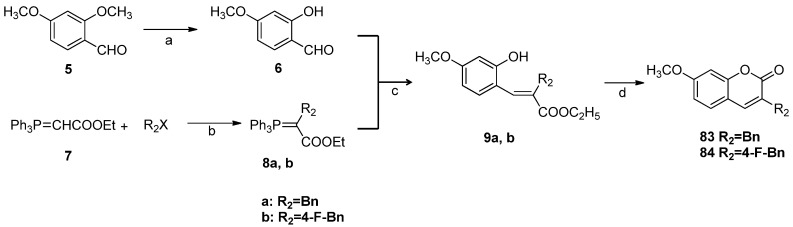
Synthesis of substituted 3-benzylcoumarin derivatives **83**–**84**^1^.

^1^*Reagents and Conditions*: (a): AlCl_3_, 1 h, r.t., 86.7%; (b): CHCl_3_, 15 h, 85 °C; (c): toluene, 15 h, 85 °C; (d): 2 h, 220 °C, nitrogen atmosphere.

**Scheme 3:**
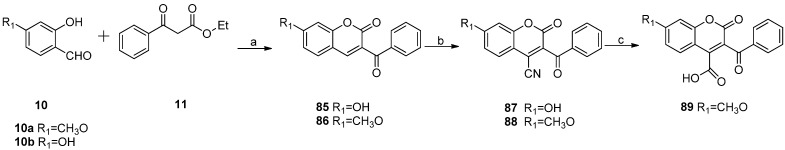
Synthesis of substituted 3-benzoyl coumarin derivatives ^1^.

^1^*Reagents and Conditions*: (a): piperidine, HCl; (b): NaCN, r.t. 1 h; I_2_, 1 h; (c): 50% H_2_SO_4_, 100 °C, 15 h.

The synthetic routes to the 3-benzyl- and 3-benzoyl coumarin derivatives are outlined in Scheme 1, Scheme 2, Scheme 3. All substituents are summarized in Table 1.

**Table 1:** Potencies in binding affinity assay. 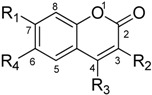

Compd.	R_1_	R_2_	R_3_	R_4_	% binding	Std	% binding	Std	IC_50_	Std (nM)
					(20 µM)		(2 µM)		(nM)	
**4b**	OH	Bn	Me	H	−6.03	0.27	−0.81	0.04		
**4c**	OH	Bn	Me	Cl	5.10	0.22	15.84	0.12		
**4d**	OH	Bn	Et	H	10.39	0.47	27.04	1.38		
**4e**	OH	Bn	*n*-Pr	H	7.06	0.16	29.06	1.28		
**4g**	OH	4-F-Bn	Et	H	19.62	0.92	10.90	0.48		
**4h**	OH	4-F-Bn	*n*-Pr	H	20.40	0.50	20.86	0.69		
**4i**	OH	H	CH_2_Cl	H	22.38	0.36	33.28	1.53		
**4j**	OH	H	CH_2_OH	H	33.58	1.64	36.28	0.56		
**12**	N(CH_3_)_2_COO	Bn	H	H	25.62	0.46	31.63	0.62		
**13**	N(CH_3_)_2_COO	Bn	Me	H	95.16	0.47	75.61	3.22	222.8	7.7
**14**	N(CH_3_)_2_COO	Bn	Me	Cl	80.76	1.80	66.26	0.74	307.3	6.9
**15**	N(CH_3_)_2_COO	Bn	Et	H	92.78	5.95	85.01	1.92	163.5	8.2
**16**	N(CH_3_)_2_COO	Bn	*n*-Pr	H	95.37	1.65	70.94	5.15	1035	68
**17**	N(CH_3_)_2_COO	4-F-Bn	Me	H	82.14	0.16	71.56	1.29	407.8	3.6
**18**	N(CH_3_)_2_COO	4-F-Bn	Et	H	99.40	3.30	98.24	5.62	54.57	1.4
**19**	N(CH_3_)_2_COO	4-F-Bn	*n*-Pr	H	87.58	2.38	66.17	1.21	157.8	10.8
**20**	N(Et)_2_COO	Bn	Me	H	20.02	0.90	15.49	0.66		
**21**	N(Et)_2_COO	Bn	Me	Cl	48.14	2.71	27.00	2.55		
**22**	N(Et)_2_COO	Bn	Et	H	48.09	1.63	6.63	1.59	6600	210
**23**	N(Et)_2_COO	Bn	*n*-Pr	H	46.11	0.43	43.14	2.78		
**24**	N(Et)_2_COO	4-F-Bn	Me	H	27.76	0.15	22.26	1.10		
**25**	N(Et)_2_COO	4-F-Bn	Et	H	59.16	2.78	25.91	3.73	2190	130
**26**	N(Et)_2_COO	4-F-Bn	*n*-Pr	H	37.50	1.25	35.17	1.46		
**27**	CH_3_COO	Bn	Me	Cl	30.83	0.66	30.55	0.77		
**28**	CH_3_COO	Bn	Me	H	96.53	0.82	72.22	3.08	519.8	11.2
**29**	CH_3_COO	Bn	Et	H	102.10	3.01	88.70	4.35	175.7	11.1
**30**	CH_3_COO	Bn	*n*-Pr	H	25.60	0.89	15.47	3.45		
**31**	CH_3_COO	4-F-Bn	Me	H	78.18	0.75	72.20	3.59	247.6	10.1
**32**	CH_3_COO	4-F-Bn	Et	H	100.96	2.00	94.57	1.06	91.2	7.9
**33**	CH_3_COO	4-F-Bn	*n*-Pr	H	72.16	3.49	61.41	3.90	252.1	7.5
**34**	EtCOO	Bn	Me	Cl	72.37	0.30	70.05	0.30	325.5	12.6
**35**	EtCOO	Bn	Me	H	85.02	3.73	64.55	3.93	1033	79
**36**	EtCOO	Bn	Et	H	87.02	3.71	81.04	0.83	391.1	8.8
**37**	EtCOO	Bn	*n*-Pr	H	38.80	1.86	19.36	2.40		
**38**	EtCOO	4-F-Bn	Me	H	90.85	1.95	75.69	4.53	189.9	12.2
**39**	EtCOO	4-F-Bn	Et	H	76.31	3.28	75.42	3.13	140.4	10.7
**40**	EtCOO	4-F-Bn	*n*-Pr	H	78.53	0.50	72.18	2.40	175.6	11.3
**41**	*n*-PrCOO	Bn	Me	Cl	25.79	0.34	20.97	0.97		
**42**	*n*-PrCOO	Bn	Me	H	68.43	1.74	53.76	3.25	352.9	12.3
**43**	*n*-PrCOO	Bn	Et	H	54.72	1.51	45.04	3.28	473.3	13.4
**44**	*n*-PrCOO	Bn	*n*-Pr	H	38.86	2.22	13.97	0.77		
**45**	*n*-PrCOO	4-F-Bn	Me	H	72.35	4.52	45.34	0.29	824.5	10.5
**46**	*n*-PrCOO	4-F-Bn	Et	H	96.49	3.67	76.94	2.67	908.6	28
**47**	*n*-PrCOO	4-F-Bn	*n*-Pr	H	70.28	6.06	56.96	2.35	944.1	10.9
**48**	BuCOO	Bn	Me	H	21.23	0.83	18.83	0.37		
**49**	BuCOO	Bn	Me	Cl	20.33	0.07	8.15	0.40		
**50**	BuCOO	Bn	Et	H	43.18	0.27	37.25	0.10		
**51**	BuCOO	Bn	*n*-Pr	H	42.48	0.12	29.52	0.97		
**52**	BuCOO	4-F-Bn	Et	H	34.02	0.89	42.77	0.31		
**53**	BuCOO	4-F-Bn	Me	H	38.28	1.30	34.74	1.63		
**54**	BuCOO	4-F-Bn	*n*-Pr	H	23.93	1.97	11.72	0.44		
**55**	*t*-BuCOO	Bn	Me	H	14.78	0.29	14.62	0.24		
**56**	*t*-BuCOO	Bn	Me	Cl	34.11	1.82	16.56	0.57		
**57**	*t*-BuCOO	Bn	Et	H	21.02	0.90	6.61	0.82		
**58**	*t*-BuCOO	Bn	*n*-Pr	H	19.05	0.83	43.16	1.78		
**59**	*t*-BuCOO	4-F-Bn	Et	H	23.36	0.12	45.92	0.65		
**60**	*t*-BuCOO	4-F-Bn	Me	H	20.26	0.78	23.71	0.06		
**61**	*t*-BuCOO	4-F-Bn	*n*-Pr	H	40.92	1.79	31.62	0.48		
**62**	PhCOO	Bn	Me	H	31.11	1.73	4.07	0.06		
**63**	PhCOO	Bn	Me	Cl	27.96	0.66	33.33	1.58		
**64**	PhCOO	Bn	Et	H	21.54	0.40	10.11	1.54		
**65**	PhCOO	Bn	*n*-Pr	H	37.15	0.59	32.57	0.68		
**66**	PhCOO	4-F-Bn	Me	H	30.96	1.14	25.78	0.02		
**67**	PhCOO	4-F-Bn	Et	H	21.28	0.70	44.56	1.26		
**68**	PhCOO	4-F-Bn	*n*-Pr	H	45.57	0.58	26.15	0.45		
**69**	PhSO_3_	Bn	Me	H	2.32	0.00	-11.27	0.42		
**70**	PhSO_3_	Bn	Me	Cl	24.53	0.39	29.87	0.08		
**71**	PhSO_3_	Bn	Et	H	−2.35	0.11	31.12	1.30		
**72**	PhSO_3_	Bn	*n*-Pr	H	14.03	0.10	26.00	1.13		
**73**	PhSO_3_	4-F-Bn	Et	H	7.40	0.31	16.59	0.56		
**74**	PhSO_3_	4-F-Bn	Me	H	17.00	0.95	21.39	0.43		
**75**	PhSO_3_	4-F-Bn	*n*-Pr	H	13.78	0.29	21.55	0.38		
**76**	TsO	Bn	Me	H	−2.36	0.03	2.87	0.09		
**77**	TsO	Bn	Me	Cl	29.57	1.22	31.68	1.30		
**78**	TsO	Bn	Et	H	4.76	0.16	33.20	1.65		
**79**	TsO	Bn	*n*-Pr	H	11.73	0.53	24.60	0.18		
**80**	TsO	4-F-Bn	Et	H	16.54	0.07	33.06	0.56		
**81**	TsO	4-F-Bn	Me	H	9.55	0.20	8.05	0.10		
**82**	TsO	4-F-Bn	*n*-Pr	H	45.87	1.77	37.51	0.33		
**83**	CH_3_O	Bn	H	H	2.93	0.05	23.04	0.00		
**84**	CH_3_O	4-F-Bn	H	H	15.43	0.51	17.56	0.68		
**85**	OH	PhCO	H	H	20.52	0.44	23.01	0.85		
**86**	CH_3_O	PhCO	H	H	−8.83	0.42	7.96	0.16		
**87**	OH	PhCO	CN	H	30.03	1.87	31.91	1.87		
**88**	CH_3_O	PhCO	CN	H	22.90	1.50	32.41	0.21		
**89**	CH_3_O	PhCO	COOH	H	6.34	0.24	16.51	0.76		

The substituted 3-benzylcoumarin derivatives have been synthesized in three steps (Scheme 1) starting from ethyl acylacetate **1** and benzyl bromide or 4-fluorobenzyl bromide in the presence of sodium hydride or potassium carbonate in DMF at 60 °C to afford the corresponding substituted ethyl acylacetates **2**. However, 2-formoxyl-ethyl-3-phenyl propionate (**2g**) can’t be obtained by this method since the starting material (ethyl formyl acetate) is not commercially available. In this case, **2g** was obtained by an alternative Claisen condensation of ethyl-3-phenyl propionate (**3**) and ethyl formate. Next, the condensation of **2** with resorcinol by the Pechmann reaction in the presence of 70% H_2_SO_4_ led to the formation of the intermediate 7-hydroxycoumarin cyclization products **4**, some of which (compounds **4b**–**e**, **4g**–**j**) were also evaluated as target compounds. Esterification of 4 with acyl chlorides in the presence of NaH afforded the target products **12**–**82** [24].

The Pechmann reaction was not suitable for the synthesis of 3-benzyl-7-*N*,*N*-dimethylcarbamoyloxyl-2*H*-chromen-2-one (**12**) which has no substituent group at the C4 position, and the overall yield of **12** was as low as 3.6%. To improve the synthetic yield of this kind of compounds, the Wittig reaction [25] was adopted and a better synthetic route was used, which provided 3-benzyl-7-methoxy-2*H*-chromen-2-one (**83**) and 3-(4-fluorobenzyl)-7-methoxy-2*H*-chromen-2-one (**84**) in four steps (Scheme 2). First, selective demethylation of 2,4-dimethoxybenzaldehyde (**5**) was performed in the presence of anhydrous AlCl_3_ to afford 2-hydroxy-4-methoxybenzaldehyde (**6**). Ethoxycarbonylmethylene triphenylphosphorane (**7**) was mixed with benzyl bromide or 4-fluorobenzyl bromide in dry chloroform to form the stable alkylated phosphorane **8**. Next **6** was reacted in toluene with phosphorane **8**, which is a resonance stabilized ylide, to yield the corresponding hydroxyesters **9**. Only *E*-isomers of **9** were obtained [25]. These were then converted thermally under nitrogen atmosphere to the respective coumarins **83** and **84** in high yields.

The substituted 3-benzoylcoumarins have been synthesized as described in Scheme 3. Condensation of *o*-hydroxybenzaldehyde (**10**) and ethyl benzoylacetate (**11**) in the presence of piperidine afforded 3-benzoyl coumarin rings **85**, **86** [26]. Compound **86** could be synthesized without solvent, while acetonitrile was required as solvent for the synthesis of **85**. In addition, it took 24 h to get **85** while the synthesis of **86** was finished almost instantaneously with the product obtained as solid, and by-products would be produced if the reaction time was prolonged. Next, cyanation occured in the presence of NaCN at room temperature for 1 h, followed by addition of iodine into the mixture and reaction for another hour to give the nitriles **87**, **88** [27]. Hydrolysis of the nitriles with 50% H_2_SO_4_ gave the corresponding 4-carboxylic acids **89** [28]. The products were purified by flash chromatography. The structures were confirmed by ^1^H and ^13^C-NMR spectroscopy, EI-MS or EI-HRMS spectra (for details, see Experimental).

### 2.3. Binding to Phosphorylated MEK1

All of the newly synthesized compounds were first tested for binding affinity to phosphorylated MEK1 at 20 μM 2 μM in a biochemical homogeneous time-resolved fluorescence (HTRF) assay. The IC_50_ values were determined for the 25 compounds with >45% inhibition of the tracer binding at 20 μM and the best IC_50_ value (54.57 nM) was observed for **18** (Table 1).

Only coumarin derivatives with benzyl- or 4-fluorobenzyl substitutions at the C3 position showed activity (compounds **12**–**84**), while all 3-benzoyl-substituted analogues demonstrated less than 40% binding affinity at 20 μM (compounds **85**–**89**). This might related to the reduced flexibility of the carbonyl group, which can block the phenyl ring from entering into the lipophilic pocket, compared with the CH_2_ in the benzyl substitution. In addition, 4-fluorobenzyl substitution at the C3 position seemed to be better than a benzyl group, as observed by comparison of **15**, **16**, **28**–**30**, **35**–**37** with their respective analogues **18**, **19**, **31**–**33**, **38**–**40**, which suggested that fluorine could enhance the binding affinity between the inhibitor and protein. Fluorine is different from other halogens. As a small atom of high electronegativity, fluorine prefers to orient towards the electropositive guanidinium side chain of Arg234 and undergoes C-F···H-N interactions, and therefore strengthens protein-ligand binding affinity, while no other halogen could have such an effect [29,30,31]. Compounds with alkyl chains, including methyl, ethyl and propyl groups, at the C4 position displayed good affinity and the ethyl group is the best of them (**13**, IC_50_ = 222.8 nM; **15**, IC_50_ = 163.5 nM; **16**, IC_50_ = 1,035 nM) while compounds without any substitution at this site (e.g., **12**) led to the disappearance of affinity. The affinities of most compounds substituted with chlorine at the C6 position (**14**, **21**, **27**, **34**, **41**, **49**, **56**, **63**, **70**, **77**) and the non-substituted analogues (**13**, **20**, **28**, **35**, **42**, **48**, **55**, **62**, **69**, **76**) displayed no obvious differences. Compounds with a *N*, *N*-dimethylcarbamoyloxyl at the C7 position displayed the best activity, and the sequence of the substituents for better binding affinity was N(CH_3_)_2_COO > MeCOO, EtCOO, *n*-PrCOO > N(Et)_2_COO, while other substituents such as *t*-BuCOO, BuCOO, PhCOO, TsO, PhSO_3_, OH, OCH_3_ showed no activity (comparing the active **18**, **25**, **32**, **39**, **46** and the inactive **52**, **59**, **67**, **73**, **80**), revealing that the affinity was quite sensitive to modifications at the 7-position. From the structures of these substituent groups at the C7 position, we speculated that their distinct activities may be related to their different steric hindrances and carbon chain lengths. Neither larger nor smaller substituents at the C7 position were good for activity. The docking results suggested that the coumarin derivatives with N(CH_3_)_2_COO, MeCOO, EtCOO, *n*-PrCOO or N(Et)_2_COO at the C7 position (compounds **18**, **25**, **32**, **39** and **46**) could occupy the lipophilic pocket suitably. Moreover, N(CH_3_)_2_COO could just occupy the pocket completely. Substituent groups whose steric hindrances and carbon chain lengths are larger than *n*-PrCOO, such as *t*-BuCOO, *n*-BuCOO, PhCOO, TsO, PhSO_3_ (compounds **52**, **59**, **67**, **73** and **80**), would extend beyond the pocket. That’s the reason why compounds with different substituents at the C7 position showed different binding affinities.

### 2.4. Inhibition of ERK Pathway

The effects of the inhibitors on the activation of ERK pathway were determined in TPA (12-*O*-tetradecanoylphorbol-13-acetate)-stimulated cells using the AP-1 luciferase reporter gene assay system. Activating protein-1 (AP-1) is the nuclear transcription factor which is a heterodimeric protein downstream of MAPK composed of dimers of the Fos and Jun proteins. The stable AP-1 dimers bind to DNA recognition elements (AP-1 response elements) and mediate transcriptional induction. AP-1 controls a number of cellular events including differentiation, proliferation and apoptosis [32,33]. Simultaneously, the expression of AP-1 can be regulated in response to different extracellular stimuli, such as cytokines, growth factors, stress, and bacterial and viral infections. ERK pathway is the most important mediator which is closely related to these responses. Activation of ERK pathway causes induction of fos gene, and then the product heterodimerizes with the Jun protein to form the AP-1 dimers. These newly formed heterodimers can enhance the expression of c-jun through AP-1 binding sites in the c-jun promoter. TPA can increase levels of AP-1 proteins through activation of the ERK pathway. U0126, a specific MEK inhibitor, can suppress the activation of the ERK pathway through inhibition of MEK1/2. It is reported that U0126 was an inhibitor of AP-1 transactivation [34]. As a cellular AP-1 antagonist, U0126 did not prevent AP-1 from DNA binding rather it inhibited the up-regulation of c-Fos and c-Jun and protein levels in activated cells. These effects resulted from inhibition of the ERK pathway. The AP-1 luciferase reporter assay system was constructed according to the effect of U0126 on AP-1 levels in TPA-activated cells as described above. This system was used to screen synthesized compounds which may have the same effect as U0126.

Twenty compounds which had excellent binding affinity with phosphorylated MEK1 were selected for further testing in intracellular assays. U0126 was taken as a positive control. In HEK 293 cells treated with TPA, 30 μM U0126 could suppress the up-regulation of AP-1 by 50%, as detected by the reporter gene assay. The AP-1 IC_50_s for inhibition of ERK pathway activation are shown in Table 2. Fifteen of 20 compounds exhibited inhibition effects on activation of the ERK pathway. Among them, six compounds (**13**, **17**, **18**, **19**, **31**, **33**) acted more efficiently than U0126 at the same concentration. All of them were 3-(4-fluorobenzyl)- or 3-benzylcoumarins with methyl, ethyl or propyl substitutions at the C4 position and *N,N*-dimethylcarbamoyloxyl or acetoxyl substitution at the C7 position. Compounds with other substituent groups at these positions showed less inhibition in this assay. 

**Table 2:** Inhibition of ERK pathway in AP-1 luciferase reporter assay.

	IC_50_ (μM)		IC_50_ (μM)
U0126	27.50 ± 1.16	**32**	N/A
**13**	5.40 ± 1.26	**33**	11.63 ± 1.20
**14**	30.80 ± 1.40	**34**	N/A
**15**	N/A	**36**	49.82 ± 1.24
**17**	4.06 ± 3.26	**38**	38.1 ± 1.26
**18**	23.37 ± 1.11	**39**	N/A
**19**	4.31 ± 2.14	**40**	87.45 ± 1.98
**20**	N/A	**42**	88.68 ± 2.52
**28**	37.56 ± 1.10	**43**	85.86 ± 1.46
**29**	90.49 ± 1.83	**67**	64.61 ± 1.74
**31**	15.70 ± 1.18		

### 2.5. Inhibition of Virus Replication

To further examine the functional effects, we selected EV71 to perform the antiviral analysis. It is established that inhibition of the ERK pathway with U0126 can suppress EV71 replication in HEK 293 and RD cells [10], so the antiviral activity of six compounds (**13**, **17**, **18**, **19**, **31**, **33**) was determined by measuring the inhibition of virus-induced cytopathic effects in cells. EC_50_s of inhibition of EV71 replication for the six compounds are summarized in Table 3. CC_50_ is the median cytotoxic concentration measured with the MTT assay. Therapeutic index (TI) was calculated as a comparison of a compound that caused the therapeutic effect (EC_50_) to the amount that caused cytotoxicity (CC_50_), whereby the higher the value of TI, the safer the compound was. The results in Table 3 showed four compounds but not **17** and **31** had inhibition effects on EV71 replication in both HEK293 and RD cells, but according to the values of TI, compounds **13** and **19** not only had a better anti-viral activity, but could also be used more safely. Examining the structures of compounds, all of them had benzyl or substituted benzyl at the C3 position, an alkyl group at the C4 position and *N,N*-dimethyl-carbamoyloxyl or acetoxyl substitution at the C7 position, suggesting that these substituent groups were beneficial to the antiviral effects.

**Table 3:** Antiviral effects in HEK293 cells and RD cells.

	HEK293 cells			RD cells		
	EC_50_ (μM)	CC_50_ (μM)	TI (CC_50_/IC_50_)	EC_50_ (μM)	CC_50_ (μM)	TI (CC_50_/IC_50_)
13	3.29	98.22	29.85	2.5	152	60.8
17	N/A	42.38	N/A	5.25	85.91	16.36
18	19.38	65.31	3.37	10	72.92	7.29
19	4.85	316.43	65.24	3.98	756.06	190
31	N/A	119.3	N/A	N/A	N/A	N/A
33	12.77	27.88	2.18	18.5	41.46	2.24

**Figure 5:**
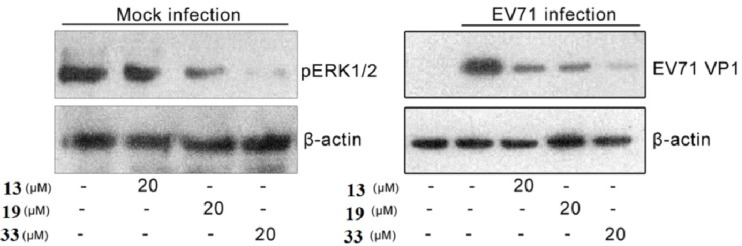
Verification of the inhibition to ERK pathway and EV71 replication in RD cells by western blots.

For compounds **13**, **19** and **33**, we did further experiments by western blots which were processed as previously described [22] to verify their inhibition to ERK pathway and EV71 replication in RD cells (Figure 5). 

The results showed that the expression of pERK and virus capsid protein VP1 can be inhibited significantly and dramatically by compounds at 20 μM. These results intuitively verified the compound inhibition of the ERK pathway and EV71 replication in RD cells.

## 3. Experimental

### 3.1. General Conditions

All solvents and reagents were used as obtained. Melting points were taken with an XT4A apparatus and are uncorrected. ^1^H-NMR and ^13^C-NMR spectra were recorded at ambient temperature using a 400 MHz Bruker Avance III spectrometer or 300 MHz JEOL JNM-AL300 spectrometer. Chemical shifts are expressed in ppm relative to tetramethylsilane. MS data were obtained with Bruker Apex IV FTMS or MDS SCIEX QSTAR systems. TLC analysises were performed on silica gel GF254 purchased from Qingdao Haiyang Chemical Co. (Qingdao, China) or Merck (Darmstadt, Germany). 

### 3.2. Chemistry

#### 3.2.1. General Procedure for the Preparation of Ethyl 2-Benzylacylacetate or Ethyl 2-(*p*-Fluorobenzyl) Acylacetates **2a**–**f**

NaH (1.2 equiv.) was added to a solution of ethyl acylacetate (**1**, 1.0 equiv.) in THF (3 mL/mmol ethyl acylacetate) at 0 °C under a nitrogen atmosphere. The reaction mixture was stirred for 15 minutes and benzyl bromide or 4-fluorobenzyl bromide (1.1 equiv.) was added dropwise. The reaction mixture was then heated to 60 °C for 12 h. The reaction mixture was poured into ice-cold saturated NH_4_Cl and extracted with EtOAc three times. The combined organic extract was washed with brine, dried over MgSO_4_ and the solvent was removed under reduced pressure. The products were purified by flash column chromatography.

*Ethyl 2-benzyl-3-oxobutanoate* (**2a**). Yield: 7.17 g, 65.1%, yellow oil. ^1^H-NMR (300 MHz, CDCl_3_): δ 1.18 (t, *J* = 7.1 Hz, 3H, CH_3_), 2.17 (s, 3H, CH_3_), 3.15 (d, *J* = 7.5 Hz, 2H, PhCH_2_), 3.78 (t, *J* = 7.5 Hz, 1H, CH), 4.13 (q, *J* = 7.2 Hz, 2H, CH_2_CH_3_), 7.16–7.28 (m, 5H, PhH).

*Ethyl 2-benzyl-3-oxopentanoate* (**2b**). Yield: 0.799 g, 68.11%, colorless oil. ^1^H-NMR (CDCl_3_): δ 1.00 (t, *J* = 7.2 Hz, 3H, COCH_2_CH_3_), 1.19 (t, *J* = 7.2 Hz, 3H, OCH_2_CH_3_), 1.49–1.59 (m, 1H, CH_2_Ph), 2.53–2.60 (m, 1H, CH_2_Ph), 3.16 (q, *J* = 7.6 Hz, 2H, COCH_2_CH_3_), 3.76–3.81 (dt, *J* = 2.4, 5.2 Hz, 1H, CHCH_2_Ph), 4.13 (q, *J* = 7.2 Hz, 2H, OCH_2_CH_3_), 7.15–7.28 (m, 5H, PhH). MS (ESI): *m/z* 235.1 (M+H^+^), 257.1 (M+Na^+^), 273.1 (M+K^+^).

*Ethyl 2-benzyl-3-oxohexanoate* (**2c**). Yield: 0.949 g, 76.4%, colorless oil. ^1^H-NMR (CDCl_3_): δ 0.84 (t, *J* = 7.2 Hz, 3H, CH_2_CH_2_CH_3_), 1.19 (t, *J* = 7.2 Hz, 3H, OCH_2_CH_3_), 1.54 (sext, *J* = 7.2 Hz, 2H, CH_2_CH_2_CH_3_), 2.27–2.35 (dt, *J* = 7.2 Hz, 1H, CH_2_Ph), 2.47–2.55 (dt, *J* = 7.2 Hz, 1H, CH_2_Ph), 3.15 (t, *J* = 7.2 Hz, 2H, CH_2_C_2_H_5_), 3.78 (t, *J* = 7.6 Hz, 1H, CHCH_2_Ph), 4.13 (q, *J* = 7.2 Hz, 2H, OCH_2_CH_3_), 7.16–7.28 (m, 5H, PhH). MS (ESI): *m/z* 249.1 (M+H^+^), 271.1 (M+Na^+^), 287.1 (M+K^+^).

*Ethyl 2-(4-fluorobenzyl)-3-oxobutanoate* (**2d)**. Yield: 1.41 g, 59%, yellow oil. ^1^H-NMR (CDCl_3_): δ 1.21 (t, *J* = 7.2 Hz, 3H, CH_2_CH_3_), 2.19 (s, 3H, CH_3_CO), 3.13 (dd, *J* =1.2, 7.2 Hz, 2H, CH_2_Ph), 3.73 (t, *J* = 7.6 Hz, 1H, CH), 4.14 (ddd, *J* = 1.2, 7.2, 11.6 Hz, 2H, CH_2_CH_3_), 6.93–6.98 (m, 2H, PhH), 7.12–7.16 (m, 2H, PhH).

*Ethyl 2-(4-fluorobenzyl)-3-oxopentanoate* (**2e**). Yield: 0.52 g, 21%, colorless oil. ^1^H-NMR (CDCl_3_): δ 1.00 (t, *J* = 7.2 Hz, 3H, CH_2_CH_3_), 1.20 (t, *J* = 7.2 Hz, 3H, OCH_2_CH_3_), 2.31–2.39 (dt, *J* = 7.2 Hz, 1H, CH_2_Ph), 2.53–2.61 (dt, *J* = 7.2 Hz, 1H, CH_2_Ph), 3.12 (q, *J* = 4.8 Hz, 2H, CH_2_CH_3_), 3.74 (t, *J* = 7.6 Hz, 1H, CHCH_2_Ph), 4.14 (dd, *J* = 7.2 Hz, 2H, OCH_2_CH_3_), 6.93–6.97 (m, 2H, PhH), 7.11–7.15 (m, 2H, PhH). MS (ESI): *m/z* 253.1 (M+H^+^), 275.1 (M+Na^+^), 291.1 (M+K^+^).

*Ethyl 2-(4-fluorobenzyl)-3-oxohexanoate* (**2f**). Yield: 2.086 g, 78.3%, colorless oil. ^1^H-NMR (CDCl_3_): δ 0.85 (t, *J* = 7.2 Hz, 3H, CH_2_CH_2_CH_3_), 1.21 (t, *J* = 7.2 Hz, 3H, OCH_2_CH_3_), 1.55 (sext, *J* = 7.2 Hz, 2H, CH_2_CH_2_CH_3_), 2.28–2.36 (dt, *J* = 7.2 Hz, 1H, CH_2_Ph), 2.48–2.56 (dt, *J* = 7.2 Hz, 1H, CH_2_Ph), 3.07–3.17 (m, 2H, CH_2_C_2_H_5_), 3.73 (t, *J* = 7.6 Hz, 1H, CHCH_2_Ph), 4.11–4.17 (dd, *J* = 7.2 Hz, 2H, OCH_2_CH_3_), 6.93–6.97 (m, 2H, PhH), 7.11–7.15 (m, 2H, PhH). MS (ESI): *m/z* 267.1 (M+H^+^), 289.1 (M+Na^+^), 305.1 (M+K^+^).

#### 3.2.2. General Procedure for the Preparation of 7-Hydroxy-2*H*-chromen-2-one derivatives **4b**–**i**

The compounds **2 **obtained above (1.0 equiv.) and resorcinol (1.0 equiv.) were suspended in 70% sulfuric acid (2 mL/mmol resorcinol) at room temperature. The reaction mixture was stirred for 12 h and poured into ice water and extracted with EtOAc three times. The combined organic extract was washed with brine, dried over MgSO_4_ and the solvent was removed under reduced pressure. The crude products were recrystalized from ethanol to afford the products.

*3-Benzyl-7-hydroxy-4-methyl-2H-chromen-2-one* (**4b**). Yield: 0.84 g, 63.2%, yellow solid, m.p.: 228–230 °C. ^1^H-NMR (DMSO-*d*_6_): δ 2.40 (s, 3H, CH_3_), 3.93 (s, 2H, CH_2_), 6.72 (d, *J* = 2.4 Hz, 1H, PhH), 6.82 (dd, *J* = 2.4, 8.8 Hz, 1H, PhH), 7.16–7.30 (m, 5H, PhH), 7.64 (d, *J* = 8.8 Hz, 1H, PhH).

*3-Benzyl-6-chloro-7-hydroxy-4-methyl-2H-chromen-2-one* (**4c**). Yield: 0.652 g, 43.5%, white powder, m.p.: 261–263 °C. ^1^H-NMR (DMSO-*d*_6_): δ 2.40 (s, 3H, CH_3_), 3.93 (s, 2H, CH_2_), 6.89 (s, 1H, PhH), 7.15–7.28 (m, 5H, PhH), 7.79 (s, 1H, PhH), 11.26 (s, 1H, OH).

*3-Benzyl-4-ethyl-7-hydroxy-2H-chromen-2-one* (**4d**). Yield: 0.251 g, 56.3%, yellow solid, m.p.: 140–141 °C. ^1^H-NMR (DMSO-*d*_6_): δ 1.02 (t, *J* = 7.6 Hz, 3H, CH_3_), 2.82 (q, *J* = 7.6 Hz, 2H, CH_2_CH_3_), 3.91 (s, 2H, CH_2_Ph), 6.72 (d, *J* = 2.8 Hz, 1H, H-8), 6.80–6.83 (dd, *J* = 2.4, 8.8 Hz, 1H, H-6), 7.17–7.29 (m, 5H, PhH), 7.64 (d, *J* = 8.8 Hz, 1H, H-5), 10.45 (s, 1H, OH). MS (ESI): *m/z* 281.1 (M+H^+^), 303.1 (M+Na^+^), 319.1 (M+K^+^).

*3-Benzyl-7-hydroxy-4-propyl-2H-chromen-2-one* (**4e**). Yield: 0.185 g, 46%, white solid, m.p.: 139–140 °C. ^1^H-NMR (DMSO-*d*_6_): δ 0.95 (t, *J* = 7.2 Hz, 3H, CH_3_), 1.35–1.42 (m, *J* = 7.6 Hz, 2H, CH_2_CH_2_CH_3_), 2.77 (t, *J* = 8.0 Hz, 2H, CH_2_C_2_H_5_), 3.91 (s, 2H, CH_2_Ph), 6.71 (d, *J* = 2.4 Hz, 1H, H-8), 6.80–6.83 (dd, *J* = 2.0, 8.8 Hz, 1H, H-6), 7.15–7.28 (m, 5H, PhH), 7.63 (d, *J* = 8.8 Hz, 1H, H-5), 10.44 (s, 1H, OH). ^13^C-NMR (DMSO-*d*_6_): δ 14.57, 22.82, 30.77, 32.33, 102.60, 111.89, 113.54, 120.11, 126.48, 127.13, 128.35, 128.83, 140.22, 152.80, 154.37, 160.84, 162.10. HRMS (ESI): *m/z* 295.13330 (M+H^+^), 317.11525 (M+Na^+^), 333.08918 (M+K^+^). 

*3-(4-Fluorobenzyl)-7-hydroxy-4-methyl-2H-chromen-2-one* (**4f**). Yield: 0.762 g, 78%, white powder, m.p.: 243–244 °C. ^1^H-NMR (DMSO-*d*_6_): δ 2.40 (s, 3H, CH_3_), 3.90 (s, 2H, CH_2_), 6.71 (s, 1H, PhH), 6.81 (d, *J* = 8.4 Hz, 1H, PhH), 7.01–7.10 (m, 2H, PhH), 7.23–7.27 (m, 2H, PhH), 7.63–7.66 (d, *J* = 8.8 Hz, 1H, PhH), 10.46 (s, 1H, OH). ^13^C-NMR (CDCl_3_): δ 15.06, 31.26, 101.89, 112.38, 112.92, 114.92, 115.13, 120.01, 126.77, 129.72, 129.80, 135.53, 148.58, 153.43, 160.46, 161.29, 161.86.

*4-Ethyl-3-(4-fluorobenzyl)-7-hydroxy-2H-chromen-2-one* (**4g**). Yield: 0.183 g, yield 31%. M.p.: 131–132 °C. Light yellow solid. ^1^H-NMR (DMSO-*d*_6_): δ 1.03 (t, *J* = 7.6 Hz, 3H, CH_3_), 2.83 (q, *J* = 7.6 Hz, 2H, CH_2_CH_3_), 3.90 (s, 2H, CH_2_Ph), 6.73 (s, 1H, H-8), 6.82 (d, *J* = 8.8 Hz, 1H, H-6), 7.09 (t, *J* = 8.8 Hz, 2H, PhH), 7.25 (t, 2H, PhH), 7.65 (d, *J* = 8.8 Hz, 1H, H-5), 10.47 (s, 1H, OH). ^13^C-NMR (DMSO-*d*_6_): δ 13.47, 21.52, 30.87, 102.18, 111.01, 113.11, 114.91, 115.12, 119.12, 126.55, 129.62, 129.70, 135.76, 153.85, 153.99, 159.45, 160.41, 161.61. HRMS (ESI): *m/z* 299.10780 (M+H^+^), 321.09033 (M+Na^+^), 337.06455 (M+K^+^).

*3-(4-Fluorobenzyl)-7-hydroxy-4-propyl-2H-chromen-2-one* (**4h**). Yield: 0.631 g, 67.4%, white solid, m.p.: 130–131 °C. ^1^H-NMR (CDCl_3_): δ 1.04 (t, *J* = 7.2 Hz, 3H, CH_2_CH_2_CH_3_), 1.52 (sext, *J* = 7.2 Hz, 2H, CH_2_CH_2_CH_3_), 2.75–2.90 (t, *J* = 8.0 Hz, 2H, CH_2_C_2_H_5_), 3.97 (s, 2H, CH_2_Ph), 6.80–6.83 (dd, *J* = 2.4, 8.8 Hz, 1H, H-6), 6.92–7.00 (m, 3H, PhH, H-8), 7.19–7.23 (m, 2H, PhH), 7.48 (d, *J* = 8.8 Hz, 1H, H-5). MS (ESI): *m/z* 313.1 (M+H^+^), 335.1 (M+Na^+^), 351.1 (M+K^+^).

*4-(Chloromethyl)-7-hydroxy-2H-chromen-2-one* (**4i**). Yield: 1.518 g, 72%, grey solid, m.p.: 166–167 °C. ^1^H-NMR (DMSO-*d*_6_): δ 4.96 (s, 2H, CH_2_), 6.42 (s, 1H, H-3), 6.76 (s, 1H, H-8), 6.85 (d, *J* = 8.8 Hz, 1H, H-6), 7.68 (d, *J* = 8.8 Hz, 1H, H-5), 10.66 (s, 1H, OH). ^13^C-NMR (DMSO-*d*_6_): δ 41.33, 102.49, 109.32, 111.03, 113.05, 126.52, 150.95, 155.27, 160.11, 161.43. HRMS (ESI): *m/z* 211.01561 (M+H^+^), 232.99758 (M+Na^+^).

#### 3.2.3. Procedure for the Preparation of 7-Hydroxy-4-(hydroxymethyl)-2H-chromen-2-one (**4j**)

Compound **4i** (1 mmol, 0.21 g) was suspended in water (20 mL) and refluxed for four days. After extraction with EtOAc three times the organic extracts were combined, washed with brine and dried over MgSO_4_. The products were purified by flash column chromatography to afford a yellow solid. Yield: 78 mg, yield 41%, m.p.: 148–149 °C. ^1^H-NMR (DMSO-*d*_6_): δ 4.71 (s, 2H, CH_2_), 5.59 (br s, 1H, ArOH), 6.24 (s, 1H, H-3), 6.73 (s, 1H, H-8), 6.78 (d, *J* = 8.4 Hz, 1H, H-6), 7.53 (d, *J* = 8.4 Hz, 1H, H-5), 10.52 (s, 1H, CH_2_OH). ^13^C-NMR (DMSO-*d*_6_): δ 59.04, 102.26, 106.51, 109.53, 112.79, 125.45, 154.83, 156.77, 160.60, 160.95. HRMS (ESI): *m/z* 193.04956 (M+H^+^), 215.03145 (M+Na^+^), 231.00538 (M+K^+^).

#### 3.2.4. Procedure for the Preparation of 3-benzyl-7-N,N-Dimethylcarbamate-2H-chromen-2-one (**12**)

To the solution of ethyl-3-phenylpropionate (**3**, 0.98 g, 5.5 mmol,1 equiv.,) in DMF (4 mL), was added NaH (0.25 g, 8.25 mmol, 1.5 equiv.) at 0 °C under a nitrogen atmosphere. The reaction mixture was stirred for 1 h and ethyl formate (0.68 mL, 8.25 mmol, 1.5 equiv.) was added. The reaction mixture was stirred for another hour at 0 °C and then 24 h at r.t. Then the pH was adjusted to 2–3 with 3 M HCl, followed by routine workup. Purification of the residue by flash column chromatography (PE–EA = 30:1) afforded 2-formoxylethyl-3-phenylpropionate (**2g**) as a yellow oil (0.718 g, 63.3%).

Compound **2g** (0.206 g, 1 mmol, 1 equiv.) and resorcinol (0.110 g, 1 mmol, 1 equiv.) were suspended in 70% sulfuric acid (4 mL), and heated at 100 °C for 1 h. The mixture was processed by routine procedures. Purification of the residue by flash column chromatography (PE–EA = 5:1) afforded 3-benzyl-7-hydroxylcoumarin (**4a**) as a thick white solid (0.074 g, 29.2%). 

To the solution of compound **4a** (0.25 g, 1 mmol, 1 equiv.) in DMF (12 mL) was added NaH (0.045 g, 1.5 mmol, 1.5 equiv.) at room temperature under a nitrogen atmosphere. The reaction mixture was stirred for 1 h and dimethycarbamoyl chloride (0.14 mL, 1.5 mmol, 1.5 equiv.) was added. The reaction mixture was stirred for another 4 h. The mixture was processed by routine procedures, then the crude product was recrystallised from ethanol as a white solid (0.063 g, 19.5%), m.p.: 151–153 °C. ^1^H-NMR (CDCl_3_): δ 3.02 (s, 3H, NCH_3_), 3.11 (s, 3H, NCH_3_), 3.88 (s, 2H, CH_2_), 7.01–7.04 (dd, *J* = 2, 8.4 Hz, 1H, PhH), 7.10 (d, *J* = 6 Hz, 1H, PhH), 7.24–7.37 (m, 7H, PhH). HRMS (ESI): *m/z* 324.12341 (M+H^+^), 346.10524 (M+Na^+^).

#### 3.2.5. General Procedure for the Preparation of 7-Carboxylate-2*H*-chromen-2-one derivatives **13**–**82** [24]

To the solution of the 7-hydroxycoumarin derivatives **4** obtained above (1.0 equiv.) in DMF (5.5 mL/mmol) was added NaH (2.0 equiv.) at room temperature under a nitrogen atmosphere. The reaction mixture was stirred for 30 minutes and acylchloride (2.0 equiv.) was added. The reaction mixture was stirred for another hour and poured into saturated NaHCO_3_. After extraction with EtOAc three times the organic extract was combined washed with brine and dried over MgSO_4_. The products were purified by flash column chromatography.

*3-Benzyl-4-methyl-2-oxo-2H-chromen-7-yl dimethylcarbamate* (**13**). Yield: 0.44 g, 86.8%, white solid, m.p.: 130–133 °C. ^1^H-NMR (CDCl_3_): δ 2.43 (s, 3H, CH_3_), 3.03 (s, 3H, NCH_3_), 3.12 (s, 3H, NCH_3_), 4.06 (s, 2H, CH_2_), 7.08–7.29 (m, 5H, PhH), 7.58 (d, *J* = 8.8 Hz, 1H, PhH). ^13^C-NMR (CDCl_3_): δ 15.48, 32.95, 36.56, 36.82, 110.10, 117.88, 118.22, 124.38, 125.15, 126.34, 128.26, 128.57, 138.82, 147.13, 152.91, 153.41, 153.99, 161.79. HRMS (ESI): *m/z* 338.13939 (M+H^+^).

*3-Benzyl-6-chloro-4-methyl-2-oxo-2H-chromen-7-yl dimethylcarbamate* (**14**). Yield: 0.193 g, 52%, white solid, m.p.: 156–157 °C. ^1^H-NMR (CDCl_3_): δ 2.42 (s, 3H, CH_3_), 3.05 (s, 3H, NCH_3_), 3.17 (s, 3H, NCH_3_), 4.06 (s, 2H, CH_2_), 7.19–7.29 (m, 6H, PhH), 7.64 (s, 1H, PhH). ^13^C-NMR (CDCl_3_): δ 15.53, 33.04, 36.64, 36.98, 112.58, 119.13, 123.18, 125.36, 125.64, 126.47, 128.24, 128.64, 138.44, 146.02, 149.19, 151.16, 152.99, 161.22. HRMS (ESI): *m/z* 372.10060 (M+H^+^). 

*3-Benzyl-4-ethyl-2-oxo-2H-chromen-7-yl dimethylcarbamate* (**15**). Yield: 0.147 g, 94.6%, white solid, m.p.: 85–86 °C. ^1^H-NMR (CDCl_3_): δ 1.14 (t, *J* = 7.6 Hz, 3H, CH_3_), 2.86 (q, *J* = 7.6 Hz, 2H, CH_2_CH_3_), 3.03 (s, 3H, NCH_3_), 3.12 (s, 3H, NCH_3_), 4.04 (s, 2H, CH_2_Ph), 7.08 (d, *J* = 2.4 Hz, 1H, H-8), 7.11–7.13 (dd, *J* = 2.4, 7.6 Hz, 1H, H-6), 7.17–7.27 (m, 5H, ArH), 7.60 (d, *J* = 8.8 Hz, 1H, H-5). ^13^C-NMR (CDCl_3_): δ 13.20, 22.27, 32.54, 36.56, 36.82, 110.38, 116.66, 118.28, 123.42, 125.12, 126.33, 128.19, 128.56, 139.04, 152.68, 153.30, 153.48, 154.00, 162.11. HRMS (ESI): *m/z* 352.15437(M+H^+^), 374.13662 (M+Na^+^).

*3-Benzyl-2-oxo-4-propyl-2H-chromen-7-yl dimethylcarbamate* (**16**). Yield: 0.44 g, 86.8%, white solid, m.p.: 117–118 °C. ^1^H-NMR (DMSO-*d*_6_): δ 0.97 (t, *J* = 7.2 Hz, 3H, CH_2_CH_2_CH_3_), 1.38–1.45 (m, *J* = 7.6 Hz, 2H, CH_2_CH_2_CH_3_), 2.84 (t, *J* = 8.0 Hz, 2H, CH_2_C_2_H_5_), 2.93 (s, 3H, NCH_3_), 3.06 (s, 3H, NCH_3_), 3.97 (s, 2H, CH_2_Ph), 7.15–7.27 (m, 7H, ArH), 7.83 (d, *J* = 8.8 Hz, 1H, H-5). ^13^C-NMR (CDCl_3_): δ 14.46, 22.46, 31.15, 32.73, 36.56, 36.82, 110.33, 116.99, 118.21, 123.80, 125.22, 126.34, 128.18, 128.55, 139.08, 151.33, 153.27, 153.39, 153.99, 162.06. HRMS (ESI): *m/z* 366.17006 (M+H^+^), 388.15222 (M+Na^+^).

*3-(4-Fluorobenzyl)-4-methyl-2-oxo-2H-chromen-7-yl dimethylcarbamate* (**17**). Yield: 0.173 g, 65.3%, white powder, m.p.: 149–150 °C. ^1^H-NMR (CDCl_3_): δ 2.44 (s, 3H, CH_3_), 3.03 (s, 3H, NCH_3_), 3.12 (s, 3H, NCH_3_), 4.02 (s, 2H, CH_2_), 6.93–6.97 (m, 2H, PhH), 7.09–7.11 (m, 2H, PhH), 7.20–7.23 (m, 2H, PhH), 7.59 (d, *J* = 8.6 Hz, 1H, PhH). ^13^C-NMR (CDCl_3_): δ 15.44, 32.20, 36.56, 36.82, 110.15, 115.24, 115.45, 117.78, 118.30, 124.25, 125.19, 129.65, 129.73, 134.42, 134.45, 147.11, 152.91, 153.50, 153.96, 160.32, 161.71, 162.74. HRMS (ESI): *m/z* 356.12960 (M+H^+^).

*4-Ethyl-3-(4-fluorobenzyl)-2-oxo-2H-chromen-7-yl dimethylcarbamate* (**18**). Yield: 0.090 g, yield 68%. M.p.: 75 °C. White solid. ^1^H-NMR (CDCl_3_): δ 1.14 (t, *J* = 7.6 Hz, 3H, CH_3_), 2.85 (q, *J* = 7.6 Hz, 2H, CH_2_CH_3_), 3.02 (s, 3H, NCH_3_), 3.12 (s, 3H, NCH_3_), 3.99 (s, 2H, CH_2_Ph), 6.94 (t, *J* = 8.0 Hz, 2H, PhH), 7.09–7.14 (m, 2H, H-6, H-8), 7.20–7.23 (m, *J* = 6.4 Hz, 2H, PhH), 7.60 (d, *J* = 8.8 Hz, 1H, H-5). ^13^C-NMR (CDCl_3_): δ 13.28, 22.25, 31.77, 36.54, 36.79, 110.37, 115.17, 115.39, 116.51, 118.38, 123.16, 125.23, 129.60, 129.68, 134.72, 152.83, 153.40, 153.96, 160.27, 162.04, 162.70. HRMS (ESI): *m/z* 370.14511 (M+H^+^), 392.12716 (M+Na^+^).

*3-(4-Fluorobenzyl)-2-oxo-4-propyl-2H-chromen-7-yl dimethylcarbamate* (**19**). Yield: 0.175 g, 91.6%, white solid, m.p.: 82–84 °C. ^1^H-NMR (CDCl_3_): δ 1.03 (t, *J* = 7.6 Hz, 3H, CH_2_CH_2_CH_3_), 1.52 (sext, *J* = 7.6 Hz, 2H, CH_2_CH_2_CH_3_), 2.80 (t, *J* = 7.6 Hz, 2H, CH_2_C_2_H_5_), 3.03 (s, 3H, NCH_3_), 3.12 (s, 3H, NCH_3_), 3.99 (s, 2H, CH_2_Ph), 6.92–6.97 (m, 2H, ArH), 7.08–7.13 (m, 2H, ArH), 7.19–7.23 (m, 2H, ArH), 7.58 (d, *J* = 8.8 Hz, 1H, H-5). ^13^C-NMR (CDCl_3_): δ 14.45, 22.52, 31.12, 31.97, 36.55, 36.81, 110.35, 115.19, 115.40, 116.86, 118.32, 123.58, 125.31, 129.57, 129.65, 134.72, 151.43, 153.36, 153.96, 160.30, 161.99, 162.73. HRMS (ESI): *m/z* 384.16069 (M+H^+^), 406.14275 (M+Na^+^).

*3-Benzyl-4-methyl-2-oxo-2H-chromen-7-yl diethylcarbamate* (**20**). Yield: 0.05 g, 27%, white solid, m.p.: 109 °C. ^1^H-NMR (CDCl_3_): δ 1.20–1.29 (dt, *J* = 6.8 Hz, 6H, CH_2_CH_3_), 2.44 (s, 3H, CH_3_), 3.39–3.46 (m, *J* = 6.8 Hz, 4H, CH_2_CH_3_), 4.06 (s, 2H, CH_2_Ph), 7.09–7.12 (m, 2H, ArH), 7.19–7.26 (m, 5H, ArH), 7.59 (d, *J* = 8.4 Hz, 1H, ArH). ^13^C-NMR (CDCl_3_): δ 13.33, 14.26, 15.50, 32.96, 42.01, 42.40, 110.05, 117.78, 118.22, 124.30, 125.12, 126.34, 128.27, 128.58, 138.84, 147.16, 152.93, 153.33, 153.48, 161.82. HRMS (ESI): *m/z* 366.16971 (M+H^+^), 388.15157 (M+Na^+^), 404.12558 (M+K^+^).

*3-Benzyl-6-chloro-4-methyl-2-oxo-2H-chromen-7-yl diethylcarbamate* (**21**). Yield: 0.069 g, 58%, white solid, m.p.: 83–84 °C. ^1^H-NMR (CDCl_3_): δ 1.21–1.32 (dt, *J* = 6.4 Hz, 6H, CH_2_CH_3_), 2.41 (s, 3H, CH_3_), 3.39–3.51 (m, *J* = 6.8 Hz, 4H, CH_2_CH_3_), 4.05 (s, 2H, CH_2_Ph), 7.18–7.27 (m, 6H, ArH), 7.64 (s, 1H, H-5). ^13^C-NMR (CDCl_3_): δ 13.27, 14.15, 15.53, 33.05, 42.21, 42.59, 112.54, 119.01, 123.30, 125.38, 125.55, 126.47, 128.25, 128.64, 138.48, 146.07, 149.28, 151.16, 152.34, 161.25. HRMS (ESI): *m/z* 400.13113 (M+H^+^), 422.11302 (M+Na^+^), 438.08702 (M+K^+^).

*3-Benzyl-4-ethyl-2-oxo-2H-chromen-7-yl diethylcarbamate* (**22**). Yield: 0.118 g, 62.4%, milk white solid, m.p.: 64–65 °C. ^1^H-NMR (CDCl_3_): δ 1.15 (t, *J* = 7.6 Hz, 3H, CH_3_), 1.21–1.29 (dt, *J* = 6.8 Hz, 6H, N(CH_2_CH_3_)_2_), 2.87 (q, *J* = 7.6 Hz, 2H, CH_2_CH_3_), 3.38–3.49 (m, *J* = 6.8 Hz, 4H, N(CH_2_CH_3_)_2_), 4.05 (s, 2H, CH_2_Ph), 7.11–7.13 (m, 2H, H-6 and H-8), 7.20–7.30 (m, 5H, CH_2_Ph), 7.61 (d, *J* = 8.4 Hz, 1H, H-5). ^13^C-NMR (CDCl_3_): δ 13.26, 13.35, 14.30, 22.30, 32.54, 42.06, 43.43, 110.33, 116.54, 118.34, 123.28, 125.17, 126.34, 128.20, 128.57, 139.08, 152.81, 153.37, 153.45, 162.17. HRMS (ESI): *m/z* 380.18545 (M+H^+^), 402.16736 (M+Na^+^), 418.14141 (M+K^+^).

*3-Benzyl-2-oxo-4-propyl-2H-chromen-7-yl diethylcarbamate* (**23**). Yield: 0.099 g, 50%, white solid, m.p.: 112–113 °C. ^1^H-NMR (CDCl_3_): δ 1.02 (3H, CH_2_CH_2_CH_3_), 1.21–1.23 (6H, CH_2_CH_3_), 1.50 (2H, CH_2_CH_2_CH_3_), 2.79 (2H, CH_2_CH_2_CH_3_), 3.41 (4H, CH_2_CH_3_), 4.03 (s, 2H, CH_2_Ph), 7.08–7.24 (m, 7H, ArH), 7.56 (d, *J* = 7.2 Hz, 1H, ArH). ^13^C-NMR (CDCl_3_): δ 18.30, 19.25, 19.42, 27.45, 36.11, 37.73, 47.04, 47.42, 115.22, 121.85, 123.17, 128.71, 130.20, 131.31, 133.18, 133.52, 144.13, 156.34, 158.30, 158.39, 167.03. HRMS (ESI): *m/z* 394.20179 (M+H^+^), 416.18395 (M+Na^+^), 432.15778 (M+K^+^).

*3-(4-Fluorobenzyl)-4-methyl-2-oxo-2H-chromen-7-yl diethylcarbamate* (**24**). Yield: 0.02 g, 10%, white solid, m.p.: 101–102 °C. ^1^H-NMR (CDCl_3_): δ 1.22–1.27 (m, 6H, CH_2_CH_3_), 2.44 (s, 3H, CH_3_), 3.39–3.45 (m, 4H, CH_2_CH_3_), 4.02 (s, 2H, CH_2_Ph), 6.95 (t, *J* = 8.0 Hz, 2H, ArH), 7.10–7.12 (m, 2H, H-6 and H-8), 7.22 (m, 2H, ArH), 7.60 (d, *J* = 8.0 Hz, 1H, H-5). ^13^C-NMR (CDCl_3_): δ 13.33, 14.27, 15.48, 32.20, 42.03, 42.42, 110.09, 115.24, 115.45, 117.67, 118.32, 124.15, 125.18, 129.66, 129.74, 134.48, 147.18, 152.91, 153.30, 153.57, 160.30, 161.75, 162.73. HRMS (ESI): *m/z* 384.16108 (M+H^+^), 406.14280 (M+Na^+^), 422.11691 (M+K^+^).

*4-Ethyl-3-(4-fluorobenzyl)-2-oxo-2H-chromen-7-yl diethylcarbamate* (**25**). Yield: 0.125 g, 63%, white solid, m.p.: 74–75 °C. ^1^H-NMR (CDCl_3_): δ 1.15 (t, *J* = 7.6 Hz, 3H, CH_3_), 1.20–1.29 (dt, *J* = 6.8 Hz, 6H, N(CH_2_CH_3_)_2_), 2.86 (q, *J* = 7.6 Hz, 2H, CH_2_CH_3_), 3.38–3.48 (m, *J* = 6.8 Hz, 4H, N(CH_2_CH_3_)_2_), 4.00 (s, 2H, CH_2_Ph), 6.96 (t, *J* = 8.4 Hz, 2H, ArH), 7.10 (d, 1H, H-6), 7.13 (s, 1H, H-8), 7.22 (m, 2H, ArH), 7.60 (d, *J* = 8.4 Hz, 1H, H-5). ^13^C-NMR (CDCl_3_): δ 18.27, 19.24, 27.24, 36.79, 47.03, 47.42, 115.34, 120.20, 120.41, 121.42, 123.34, 128.16, 130.12, 134.58, 134.66, 139.67, 139.70, 157.73, 158.30, 158.47, 165.30, 167.03, 167.73. HRMS (ESI): *m/z* 398.17617 (M+H^+^), 420.15785 (M+Na^+^), 436.13206 (M+K^+^).

*3-(4-Fluorobenzyl)-2-oxo-4-propyl-2H-chromen-7-yl diethylcarbamate* (**26**). Yield: 0.134 g, 65%, white solid, mp: 51–52 °C. ^1^H-NMR (CDCl_3_): δ 1.04 (t, *J* = 7.2 Hz, 3H, CH_2_CH_2_CH_3_), 1.20–1.29 (m, 6H, CH_2_CH_3_), 1.52–1.53 (m, 2H, CH_2_CH_2_CH_3_), 2.80 (br s, 2H, CH_2_CH_2_CH_3_), 3.40–3.46 (m, 4H, CH_2_CH_3_), 4.00 (s, 2H, CH_2_Ph), 6.95 (t, *J* = 8.4 Hz, 2H, ArH), 7.10–7.14 (m, 2H, H-6 and H-8), 7.20–7.23 (m, 2H, ArH), 7.58 (d, *J* = 8.4 Hz, 1H, H-5).^13^C-NMR (CDCl_3_): δ 13.35, 14.29, 14.50, 22.57, 31.15, 32.00, 42.09, 42.45, 110.31, 115.22, 115.44, 116.79, 118.35, 123.54, 125.32, 129.64, 134.79, 151.49, 153.35, 160.34, 162.04, 162.75. HRMS (ESI): *m/z* 412.19179 (M+H^+^), 434.17324 (M+Na^+^).

*3-Benzyl-6-chloro-4-methyl-2-oxo-2H-chromen-7-yl acetate* (**27**). Yield: 0.05 g, 29%, white solid, m.p.: 190 °C. ^1^H-NMR (CDCl_3_): δ 2.39 (s, 3H, CH_3_CO), 2.43 (s, 3H, CH_3_), 4.06 (s, 2H, CH_2_Ph), 7.15 (s, 1H, H-8), 7.20–7.27 (m, 5H, CH_2_Ph), 7.68 (s, 1H, H-5). ^13^C-NMR (CDCl_3_): δ 15.56, 20.60, 33.07, 112.32, 119.73, 122.97, 125.69, 126.08, 126.53, 128.24, 128.67, 138.31, 145.88, 148.40, 151.16, 161.01, 167.94. HRMS (ESI): *m/z* 343.07294 (M+H^+^), 365.05485 (M+Na^+^).

*3-Benzyl-4-methyl-2-oxo-2H-chromen-7-yl acetate* (**28**). Yield: 0.082 g, 55%, white solid, m.p.: 165–166 °C. ^1^H-NMR (CDCl_3_): δ 2.33 (s, 3H, CH_3_CO), 2.44 (s, 3H, CH_3_), 4.06 (s, 2H, CH_2_Ph), 7.05 (dd, *J* = 8.8, 2.0 Hz, 1H, H-6), 7.10 (d, *J* = 2.0 Hz, 1H, H-8), 7.18–7.29 (m, 5H, CH_2_Ph), 7.62 (d, *J* = 8.8 Hz, 1H, H-5). ^13^C-NMR (CDCl_3_): δ 15.51, 21.11, 32.97, 110.16, 118.00, 118.47, 124.85, 125.43, 126.40, 128.25, 128.60, 138.69, 146.98, 152.34, 152.90, 161.61, 168.82. HRMS (ESI): *m/z* 309.11175 (M+H^+^), 331.09374 (M+Na^+^), 347.06770 (M+K^+^).

*3-Benzyl-4-ethyl-2-oxo-2H-chromen-7-yl acetate* (**29**). Yield: 0.11 g, 66%, white solid, m.p.: 123–125 °C. ^1^H-NMR (CDCl_3_): δ 1.14 (t, *J* = 7.6 Hz, 3H, CH_2_CH_3_), 2.34 (s, 3H, CH_3_CO), 2.86 (q, *J* = 7.6 Hz, 2H, CH_2_CH_3_), 4.04 (s, 2H, CH_2_Ph), 7.06 (d, *J* = 8.8 Hz, 1H, H-6), 7.12 (s, 1H, H-8), 7.19–7.29 (m, 5H, CH_2_Ph), 7.62 (d, *J* = 8.8 Hz, 1H, H-5). ^13^C-NMR (CDCl_3_): δ 13.20, 21.12, 22.28, 32.56, 110.43, 117.26, 118.05, 123.90, 125.39, 126.40, 128.19, 128.59, 138.91, 152.24, 152.52, 153.46, 161.91, 168.84. HRMS (ESI): *m/z* 323.12751 (M+H^+^), 345.10923 (M+Na^+^), 361.08309 (M+K^+^).

*3-Benzyl-2-oxo-4-propyl-2H-chromen-7-yl acetate* (**30**). Yield: 0.105 g, 63%, white solid, m.p.: 139–140 °C. ^1^H-NMR (CDCl_3_): δ 1.04 (t, *J* = 7.2 Hz, 3H, CH_2_CH_2_CH_3_), 1.49–1.55 (m, *J* = 7.6 Hz, 2H, CH_2_CH_2_CH_3_), 2.34 (s, 3H, CH_3_CO), 2.81 (t, *J* = 7.2 Hz, 2H, CH_2_CH_2_CH_3_), 4.04 (s, 2H, CH_2_Ph), 7.05 (d, *J* = 8.4 Hz, 1H, H-6), 7.11 (s, 1H, H-8), 7.19–7.29 (m, 5H, CH_2_Ph), 7.60 (d, *J* = 8.8 Hz, 1H, H-5). ^13^C-NMR (CDCl_3_): δ 14.48, 21.12, 22.46, 31.15, 32.75, 110.38, 117.58, 117.99, 124.28, 125.48, 126.40, 128.17, 128.58, 138.95, 151.17, 152.21, 153.38, 161.86, 168.83. HRMS (ESI): *m/z* 337.14307 (M+H^+^), 359.12512 (M+Na^+^), 375.09918 (M+K^+^).

*3-(4-Fluorobenzyl)-4-methyl-2-oxo-2H-chromen-7-yl acetate* (**31**). Yield: 0.100 g, 61%, white solid, m.p.: 119–120 °C. ^1^H-NMR (CDCl_3_): δ 2.34 (s, 3H, CH_3_CO), 2.44 (s, 3H, CH_3_), 4.02 (s, 2H, CH_2_Ph), 6.95 (t, *J* = 8.4 Hz, 2H, ArH), 7.06–7.11 (m, 2H, H-6 and H-8), 7.12 (s, 1H, H-8), 7.21–7.26 (m, 2H, ArH), 7.62 (d, *J* = 8.8 Hz, 1H, H-5). ^13^C-NMR (CDCl_3_): δ 15.47, 21.11, 32.21, 110.19, 115.26, 115.47, 118.09, 118.36, 124.69, 125.49, 129.66, 129.74, 134.31, 134.34, 146.97, 152.43, 152.88, 160.32, 161.52, 162.75, 168.80. HRMS (ESI): *m/z* 327.10315 (M+H^+^), 349.08500 (M+Na^+^), 365.05920 (M+K^+^).

*4-Ethyl-3-(4-fluorobenzyl)-2-oxo-2H-chromen-7-yl acetate* (**32**). Yield: 0.12 g, 70%, white solid, m.p.: 122–123 °C. ^1^H-NMR (CDCl_3_): δ 1.16 (t, *J* = 7.6 Hz, 3H, CH_2_CH_3_), 2.34 (s, 3H, CH_3_CO), 2.86 (q, *J* = 7.6 Hz, 2H, CH_2_CH_3_), 4.00 (s, 2H, CH_2_Ph), 6.95 (t, *J* = 8.4 Hz, 2H, ArH), 7.07 (d, *J* = 8.8 Hz, 1H, H-6), 7.12 (s, 1H, H-8), 7.20–7.26 (m, 2H, ArH), 7.63 (d, *J* = 8.8 Hz, 1H, H-5). ^13^C-NMR (CDCl_3_): δ 13.27, 21.11, 22.26, 31.82, 110.47, 115.25, 115.46, 117.15, 118.14, 123.72, 125.43, 129.59, 129.67, 134.52, 134.55, 152.33, 152.56, 153.45, 160.33, 161.83, 162.76, 168.83. HRMS (ESI): *m/z* 341.11816 (M+H^+^), 363.10008 (M+Na^+^).

*3-(4-Fluorobenzyl)-2-oxo-4-propyl-2H-chromen-7-yl acetate* (**33**). Yield: 0.127 g, 72%, white solid, m.p.: 150–151 °C. ^1^H-NMR (CDCl_3_): δ 1.07 (t, *J* = 7.6 Hz, 3H, CH_2_CH_2_CH_3_), 1.52–1.58 (m, *J* = 7.6 Hz, 2H, CH_2_CH_2_CH_3_), 2.36 (s, 3H, CH_3_CO), 2.80–2.85 (m, 2H, CH_2_CH_2_CH_3_), 4.02 (s, 2H, CH_2_Ph), 6.98 (t, *J* = 8.4 Hz, 2H, ArH), 7.07–7.10 (dd, *J* = 2.0, 8.4 Hz, 1H, H-6), 7.13 (d, *J* = 2.0 Hz, 1H, H-8), 7.21–7.25 (m, 2H, ArH), 7.63 (d, *J* = 8.8 Hz, 1H, H-5). ^13^C-NMR (CDCl_3_): δ 14.49, 21.12, 22.52, 31.14, 32.01, 110.43, 115.25, 115.46, 117.47, 118.08, 124.11, 125.54, 129.57, 129.65, 134.58, 151.20, 152.30, 153.37, 160.34, 161.78, 162.77, 168.82. HRMS (ESI): *m/z* 355.13436 (M+H^+^), 377.11631 (M+Na^+^), 393.09050 (M+K^+^).

*3-Benzyl-6-chloro-4-methyl-2-oxo-2H-chromen-7-yl propionate* (**34**). Yield: 0.047 g, 44%, white solid, m.p.: 124–126 °C. ^1^H-NMR (CDCl_3_): δ 1.32 (t, *J* = 7.2 Hz, 3H, CH_2_CH_3_), 2.43 (s, 3H, CH_3_), 2.69 (q, *J* = 7.2 Hz, 2H, CH_2_CH_3_), 4.06 (s, 2H, CH_2_Ph), 7.15 (s, 1H, H-8), 7.19–7.28 (m, 5H, CH_2_Ph), 7.67 (s, 1H, H-5). ^13^C-NMR (CDCl_3_): δ 8.99, 15.56, 27.45, 33.07, 112.33, 119.63, 123.00, 125.66, 126.01, 126.52, 128.25, 128.67, 138.34, 145.92, 148.51, 151.18, 161.05, 171.48. HRMS (ESI): *m/z* 357.08905 (M+H^+^), 379.07086 (M+Na^+^), 395.04497 (M+K^+^).

*3-Benzyl-4-methyl-2-oxo-2H-chromen-7-yl propionate* (**35**). Yield: 0.14 g, 87%, white solid, m.p.: 118–119 °C. ^1^H-NMR (CDCl_3_): δ 1.28 (t, *J* = 7.6 Hz, 3H, CH_2_CH_3_), 2.45 (s, 3H, CH_3_), 2.63 (q, *J* = 7.6 Hz, 2H, CH_2_CH_3_), 4.07 (s, 2H, CH_2_Ph), 7.06 (d, *J* = 8.4 Hz, 1H, H-6), 7.10 (s, 1H, H-8), 7.19–7.29 (m, 5H, CH_2_Ph), 7.61 (d, *J* = 8.8 Hz, 1H, H-5). ^13^C-NMR (CDCl_3_): δ 8.93, 15.50, 27.74, 32.97, 110.12, 118.00, 118.38, 124.78, 125.38, 126.39, 128.26, 128.60, 138.71, 147.00, 152.50, 152.92, 161.64, 172.35. HRMS (ESI): *m/z* 323.12765 (M+H^+^), 345.10937 (M+Na^+^), 361.08330 (M+K^+^).

*3-Benzyl-4-ethyl-2-oxo-2H-chromen-7-yl propionate* (**36**). Yield: 0.143 g, 85%, white solid, m.p.: 124 °C. ^1^H-NMR (CDCl_3_): δ 1.15 (t, *J* = 7.6 Hz, 3H, CH_2_CH_3_), 1.28 (t, *J* = 7.6 Hz, 3H, COCH_2_CH_3_), 2.63 (q, *J* = 7.6 Hz, 2H, CH_2_CH_3_), 2.87 (q, *J* = 7.6 Hz, 2H, COCH_2_CH_3_), 4.05 (s, 2H, CH_2_Ph), 7.06 (d, *J* = 8.8 Hz, 1H, H-6), 7.12 (s, 1H, H-8), 7.19–7.29 (m, 5H, CH_2_Ph), 7.62 (d, *J* = 8.8 Hz, 1H, H-5). ^13^C-NMR (CDCl_3_): δ 8.94, 13.21, 22.28, 27.74, 32.56, 110.39, 117.16, 118.06, 123.82, 125.34, 126.39, 128.20, 128.58, 138.93, 152.40, 152.54, 153.48, 161.95, 172.37. HRMS (ESI): *m/z* 337.14303 (M+H^+^), 359.12504 (M+Na^+^).

*3-Benzyl-2-oxo-4-propyl-2H-chromen-7-yl propionate* (**37**). Yield: 0.121 g, 69%, white solid, m.p.: 120–121 °C. ^1^H-NMR (CDCl_3_): δ 1.04 (t, *J* = 7.2 Hz, 3H, CH_2_CH_2_CH_3_), 1.28 (t, *J* = 7.6 Hz, 3H, COCH_2_CH_3_), 1.49–1.57 (m, *J* = 7.6 Hz, 2H, CH_2_CH_2_CH_3_), 2.63 (q, *J* = 7.6 Hz, 2H, COCH_2_CH_3_), 2.81 (t, *J* = 8.0 Hz, 2H, CH_2_CH_2_CH_3_), 4.04 (s, 2H, CH_2_Ph), 7.05 (d, *J* = 8.8 Hz, 1H, H-6), 7.11 (s, 1H, H-8), 7.19–7.29 (m, 5H, CH_2_Ph), 7.59 (d, *J* = 8.4 Hz, 1H, H-5). ^13^C-NMR (CDCl_3_): δ 8.94, 14.48, 22.47, 27.74, 31.16, 32.75, 110.34, 117.48, 117.99, 124.19, 125.44, 126.40, 128.18, 128.57, 138.97, 151.20, 152.37, 153.39, 161.90, 172.35. HRMS (ESI): *m/z* 351.15891 (M+H^+^), 373.14083 (M+Na^+^), 389.11452 (M+K^+^).

*3-(4-Fluorobenzyl)-4-methyl-2-oxo-2H-chromen-7-yl propionate* (**38**). Yield: 0.082 g, 48%, white solid, m.p.: 115–116 °C. ^1^H-NMR (CDCl_3_): δ 1.28 (t, *J* = 7.6 Hz, 3H, CH_2_CH_3_), 2.45 (s, 3H, CH_3_), 2.63 (q, *J* = 7.6 Hz, 2H, CH_2_CH_3_), 4.02 (s, 2H, CH_2_Ph), 6.95 (t, *J* = 8.0 Hz, 2H, ArH), 7.06–7.10 (m, 2H, H-6 and H-8), 7.20–7.26 (m, 2H, ArH), 7.62 (d, *J* = 8.8 Hz, 1H, H-5). ^13^C-NMR (CDCl_3_): δ 8.93, 15.47, 27.73, 32.22, 110.16, 115.26, 115.48, 118.09, 118.27, 124.63, 125.43, 129.67, 129.75, 134.33, 134.36, 146.98, 152.59, 152.91, 160.33, 161.56, 162.76, 172.33. HRMS (ESI): *m/z* 341.11829 (M+H^+^), 363.10044 (M+Na^+^), 379.07431 (M+K^+^).

*4-Ethyl-3-(4-fluorobenzyl)-2-oxo-2H-chromen-7-yl propionate* (**39**). Yield: 0.14 g, 81%, white solid, m.p.: 97–98 °C. ^1^H-NMR (CDCl_3_): δ 1.16 (t, *J* = 7.6 Hz, 3H, CH_2_CH_3_), 1.28 (t, *J* = 7.6 Hz, 3H, COCH_2_CH_3_), 2.63 (q, *J* = 7.6 Hz, 2H, CH_2_CH_3_), 2.87 (q, *J* = 7.6 Hz, 2H, COCH_2_CH_3_), 4.00 (s, 2H, CH_2_Ph), 6.95 (t, *J* = 8.4 Hz, 2H, ArH), 7.07 (d, *J* = 8.8 Hz, 1H, H-6), 7.12 (s, 1H, H-8), 7.20–7.24 (m, 2H, ArH), 7.62 (d, *J* = 8.8 Hz, 1H, H-5). ^13^C-NMR (CDCl_3_): δ 8.93, 13.27, 22.26, 27.74, 31.82, 110.43, 115.25, 115.46, 117.04, 118.14, 123.65, 125.38, 129.60, 129.68, 134.54, 134.57, 152.49, 152.57, 153.47, 160.33, 161.87, 162.76, 172.35. HRMS (ESI): *m/z* 355.13383 (M+H^+^), 377.11579 (M+Na^+^).

*3-(4-Fluorobenzyl)-2-oxo-4-propyl-2H-chromen-7-yl propionate* (**40**). Yield: 0.113 g, 61%, white solid, m.p.: 94–95 °C. ^1^H-NMR (CDCl_3_): δ 1.05 (t, *J* = 7.2 Hz, 3H, CH_2_CH_2_CH_3_), 1.28 (t, *J* = 7.6 Hz, 3H, COCH_2_CH_3_), 1.50–1.56 (m, *J* = 7.6 Hz, 2H, CH_2_CH_2_CH_3_), 2.63 (q, *J* = 7.6 Hz, 2H, COCH_2_CH_3_), 2.81 (t, *J* = 7.6 Hz, 2H, CH_2_CH_2_CH_3_), 4.00 (s, 2H, CH_2_Ph), 6.95 (t, *J* = 8.4 Hz, 2H, ArH), 7.06 (d, *J* = 8.8 Hz, 1H, H-6), 7.11 (s, 1H, H-8), 7.19–7.23 (m, 2H, ArH), 7.60 (d, *J* = 8.8 Hz, 1H, H-5). ^13^C-NMR (CDCl_3_): δ 8.93, 14.49, 22.53, 27.74, 31.14, 32.01, 110.39, 115.24, 115.45, 117.37, 118.09, 124.03, 125.49, 129.58, 129.66, 134.58, 134.61, 151.23, 152.46, 153.38, 160.34, 161.82, 162.77, 172.35. HRMS (ESI): *m/z* 369.15020 (M+H^+^), 391.13211 (M+Na^+^), 407.10587 (M+K^+^).

*3-Benzyl-6-chloro-4-methyl-2-oxo-2H-chromen-7-yl butyrate* (**41**). Yield: 0.076 g, 68.5%, white solid, m.p.: 107–108 °C. ^1^H-NMR (CDCl_3_): δ 1.07 (t, *J* = 7.2 Hz, 3H, CH_2_CH_2_CH_3_), 1.83 (m, 2H, CH_2_CH_2_CH_3_), 2.42 (s, 2H, CH_3_), 2.63 (t, *J* = 7.2 Hz, 2H, CH_2_CH_2_CH_3_), 4.05 (s, 2H, CH_2_Ph), 7.13 (s, 1H, H-8), 7.18–7.28 (m, 5H, ArH), 7.66 (s, 1H, H-5). ^13^C-NMR (CDCl_3_): δ13.65, 15.55, 18.34, 33.07, 35.84, 112.34, 119.62, 123.00, 125.67, 125.99, 126.52, 128.25, 128.66, 138.35, 145.93, 148.50, 151.16, 161.03, 170.64. HRMS (ESI): *m/z* 371.10463 (M+H^+^), 393.08669 (M+Na^+^), 409.06073 (M+K^+^).

*3-Benzyl-4-methyl-2-oxo-2H-chromen-7-yl butyrate* (**42**). Yield: 0.15 g, 89%, white solid, m.p.: 139–140 °C. ^1^H-NMR (CDCl_3_): δ 1.05 (t, *J* = 7.6 Hz, 3H, CH_2_CH_3_), 1.80 (q, *J* = 7.6 Hz, 2H, CH_2_CH_3_), 2.44 (s, 3H, CH_3_), 2.57 (t, *J* = 7.6 Hz, 2H, CH_2_CH_2_CH_3_), 4.06 (s, 2H, CH_2_Ph), 7.05 (d, *J* = 8.8 Hz, 1H, H-6), 7.09 (s, 1H, H-8), 7.19–7.26 (m, 5H, CH_2_Ph), 7.61 (d, *J* = 8.8 Hz, 1H, H-5). ^13^C-NMR (CDCl_3_): δ 13.61, 15.51, 18.34, 32.97, 36.17, 110.14, 118.04, 118.37, 124.77, 125.39, 126.39, 128.26, 128.60, 138.72, 147.00, 152.48, 152.91, 161.63, 171.52. HRMS (ESI): *m/z* 337.14308 (M+H^+^), 359.12508 (M+Na^+^).

*3-Benzyl-4-ethyl-2-oxo-2H-chromen-7-yl butyrateen-7-yl propionate* (**43**). Yield: 0.18 g, 100%, white solid, m.p.: 102 °C. ^1^H-NMR (CDCl_3_): δ 1.06 (t, *J* = 7.2 Hz, 3H, COCH_2_CH_2_CH_3_), 1.14 (t, *J* = 7.6 Hz, 3H, CH_2_CH_3_), 1.78–1.83 (m, *J* = 7.2 Hz, 2H, COCH_2_CH_2_CH_3_), 2.58 (t, *J* = 7.2 Hz, 2H, COCH_2_CH_2_CH_3_), 2.87 (q, *J* = 7.6 Hz, 2H, CH_2_CH_3_), 4.04 (s, 2H, CH_2_Ph), 7.05 (d, *J* = 8.8 Hz, 1H, H-6), 7.11 (s, 1H, H-8), 7.19–7.29 (m, 5H, CH_2_Ph), 7.62 (d, *J* = 8.8 Hz, 1H, H-5). ^13^C-NMR (CDCl_3_): δ 13.20, 13.61, 22.28, 29.71, 32.57, 36.17, 110.42, 117.15, 118.08, 123.82, 125.34, 126.38, 128.19, 128.58, 138.94, 152.38, 152.53, 153.48, 161.93, 171.55. HRMS (ESI): *m/z* 351.15891 (M+H^+^), 373.14081 (M+Na^+^), 389.11448 (M+K^+^).

*3-Benzyl-2-oxo-4-propyl-2H-chromen-7-yl butyrate* (**44**). Yield: 0.167 g, 92%, white solid, m.p.: 105–106 °C. ^1^H-NMR (CDCl_3_): δ 1.02–1.07 (m, *J* = 7.6 Hz, 6H, CH_3_), 1.49–1.55 (m, *J* = 7.6 Hz, 2H, CH_2_CH_2_CH_3_), 1.77–1.83 (m, *J* = 7.6 Hz, 2H, COCH_2_CH_2_CH_3_), 2.58 (t, *J* = 7.2 Hz, 2H, COCH_2_CH_2_CH_3_), 2.81 (t, *J* =8.0 Hz, 2H, CH_2_CH_2_CH_3_), 4.04 (s, 2H, CH_2_Ph), 7.04 (d, *J* = 8.8 Hz, 1H, H-6), 7.10 (s, 1H, H-8), 7.19–7.29 (m, 5H, CH_2_Ph), 7.59 (d, *J* = 8.4 Hz, 1H, H-5). ^13^C-NMR (CDCl_3_): δ 13.60, 14.48, 18.35, 22.47, 31.15, 32.76, 36.17, 110.37, 117.48, 118.03, 124.19, 125.45, 126.40, 128.18, 128.57, 138.98, 151.20, 152.36, 153.39, 161.89, 171.54. HRMS (ESI): *m/z* 365.17454 (M+H^+^), 387.15649 (M+Na^+^), 403.13042 (M+K^+^).

*3-(4-Fluorobenzyl)-4-methyl-2-oxo-2H-chromen-7-yl butyrate* (**45**). Yield: 0.122 g, 69%, white solid, m.p.: 85–86 °C. ^1^H-NMR (CDCl_3_): δ 1.05 (t, *J* = 7.2 Hz, 3H, CH_2_CH_2_CH_3_), 1.77–1.83 (m, *J* = 7.2 Hz, 2H, CH_2_CH_2_CH_3_), 2.45 (s, 3H, CH_3_), 2.58 (t, *J* = 7.2Hz, 2H, CH_2_CH_2_CH_3_), 4.02 (s, 2H, CH_2_Ph), 6.95 (t, *J* = 8.0 Hz, 2H, ArH), 7.05–7.10 (m, 2H, H-6 and H-8), 7.20–7.26 (m, 2H, ArH), 7.62 (d, *J* = 8.8 Hz, 1H, H-5). ^13^C-NMR (CDCl_3_): δ 13.61, 15.47, 18.34, 32.22, 36.16, 110.18, 115.26, 115.47, 118.13, 118.27, 124.63, 125.43, 129.67, 129.75, 134.33, 134.36, 146.99, 152.57, 152.90, 160.32, 161.54, 162.75, 171.51. HRMS (ESI): *m/z* 355.13422 (M+H^+^), 377.11617 (M+Na^+^), 393.09024 (M+K^+^).

*4-Ethyl-3-(4-fluorobenzyl)-2-oxo-2H-chromen-7-yl butyrate* (**46**). Yield: 0.155 g, 84%, white solid, m.p.: 79 °C. ^1^H-NMR (CDCl_3_): δ 1.06 (t, *J* = 7.6 Hz, 3H, CH_2_CH_2_CH_3_), 1.16 (t, *J* = 7.6 Hz, 3H, CH_2_CH_3_), 1.78–1.83 (m, *J* = 7.6 Hz, 2H, CH_2_CH_2_CH_3_), 2.58 (t, *J* = 7.6 Hz, 2H, CH_2_CH_2_CH_3_), 2.87 (q, *J* = 7.6 Hz, 2H, CH_2_CH_3_), 4.00 (s, 2H, CH_2_Ph), 6.95 (t, *J* = 8.4 Hz, 2H, ArH), 7.06 (d, *J* = 8.4 Hz, 1H, H-6), 7.11 (s, 1H, H-8), 7.20–7.24 (m, 2H, ArH), 7.62 (d, *J* = 8.4 Hz, 1H, H-5). ^13^C-NMR (CDCl_3_): δ 13.27, 13.60, 18.34, 22.26, 31.82, 36.17, 110.46, 115.25, 115.46, 117.04, 118.17, 123.66, 125.37, 129.59, 129.67, 134.53, 134.57, 152.47, 152.56, 153.47, 160.34, 161.85, 162.77, 171.54. HRMS (ESI): *m/z* 369.14937 (M+H^+^), 391.13124 (M+Na^+^). 

*3-(4-Fluorobenzyl)-2-oxo-4-propyl-2H-chromen-7-yl butyrate* (**47**). Yield: 0.122 g, 64%, white solid, m.p.: 88–90 °C. ^1^H-NMR (CDCl_3_): δ 1.05–1.07 (m, 6H, CH_3_), 1.50–1.56 (m, *J* = 7.6 Hz, 2H, CH_2_CH_2_CH_3_), 1.77–1.83 (m, *J* = 7.2 Hz, 2H, COCH_2_CH_2_CH_3_), 2.58 (t, *J* = 7.2 Hz, 2H, COCH_2_CH_2_CH_3_), 2.80 (t, *J* = 7.6 Hz, 2H, CH_2_CH_2_CH_3_), 4.00 (s, 2H, CH_2_Ph), 6.95 (t, *J* = 8.4 Hz, 2H, ArH), 7.05 (d, *J* = 8.8 Hz, 1H, H-6), 7.10 (s, 1H, H-8), 7.21–7.22 (m, 2H, ArH), 7.60 (d, *J* = 8.8 Hz, 1H, H-5). ^13^C-NMR (CDCl_3_): δ 13.60, 14.49, 18.35, 22.53, 31.14, 32.01, 36.17, 110.42, 115.24, 115.45, 117.37, 118.12, 124.03, 125.48, 129.58, 129.65, 134.58, 151.22, 152.44, 153.38, 160.34, 161.81, 162.77, 171.53. HRMS (ESI): *m/z* 383.16575 (M+H^+^), 405.14765 (M+Na^+^), 421.12162 (M+K^+^).

*3-Benzyl-4-methyl-2-oxo-2H-chromen-7-yl pentanoate* (**48**). Yield: 0.151 g, 86%, white solid, m.p.: 110–111 °C. ^1^H-NMR (CDCl_3_): δ 0.98 (t, *J* = 7.6 Hz, 3H, CH_2_CH_3_), 1.43–1.48 (m, *J* = 7.6 Hz, 2H, CH_2_CH_3_), 1.73–1.77 (m, *J* = 7.6 Hz, 2H, CH_2_CH_2_CH_3_), 2.44 (s, 3H, CH_3_), 2.59 (t, *J* = 7.6 Hz, 2H, COCH_2_), 4.06 (s, 2H, CH_2_Ph), 7.05 (d, *J* = 8.8 Hz, 1H, H-6), 7.09 (s, 1H, H-8), 7.18–7.25 (m, 5H, CH_2_Ph), 7.61 (d, *J* = 8.8 Hz, 1H, H-5). ^13^C-NMR (CDCl_3_): δ 13.71, 15.50, 22.23, 26.86, 32.97, 34.07, 110.13, 118.03, 118.37, 124.76, 125.39, 126.38, 128.27, 128.60, 138.73, 147.00, 152.50, 152.91, 161.62, 171.70. HRMS (ESI): *m/z* 351.15892 (M+H^+^), 373.14081 (M+Na^+^), 389.11455 (M+K^+^).

*3-Benzyl-6-chloro-4-methyl-2-oxo-2H-chromen-7-yl pentanoate* (**49**). Yield: 0.099 g, 86%, white solid, m.p.: 108–109 °C. ^1^H-NMR (CDCl_3_): δ 0.98 (t, *J* = 7.2 Hz, 3H, CH_2_CH_3_), 1.45–1.50 (m, *J* = 7.2 Hz, 2H, CH_2_CH_2_CH_2_CH_3_), 1.74–1.80 (m, *J* = 7.2 Hz, 2H, CH_2_CH_2_CH_2_CH_3_), 2.42 (s, 3H, CH_3_), 2.65 (t, *J* = 7.2 Hz, 2H, CH_2_CH_2_CH_2_CH_3_), 4.05 (s, 2H, CH_2_Ph), 7.13 (s, 1H, H-8), 7.19–7.29 (m, 5H, CH_2_PhH), 7.66 (s, 1H, H-5). ^13^C-NMR (CDCl_3_): δ 13.70, 15.55, 22.23, 26.82, 33.07, 33.72, 112.34, 119.62, 123.00, 125.67, 125.99, 126.52, 128.25, 128.66, 138.35, 145.93, 148.51, 151.16, 161.03, 170.81. HRMS (ESI): *m/z* 385.12011 (M+H^+^), 407.10215 (M+Na^+^), 423.07623 (M+K^+^).

*3-Benzyl-4-ethyl-2-oxo-2H-chromen-7-yl pentanoate* (**50**). Yield: 0.112 g, 62%, white solid, m.p.: 72–73 °C. ^1^H-NMR (CDCl_3_): δ 0.98 (t, *J* = 7.2 Hz, 3H, CH_2_CH_2_CH_2_CH_3_), 1.14 (t, *J* = 6.8 Hz, 3H, CH_2_CH_3_), 1.43–1.48 (m, *J* = 6.8 Hz, 2H, CH_2_CH_2_CH_2_CH_3_), 1.74–1.77 (m, *J* = 6.8 Hz, 2H, CH_2_CH_2_CH_2_CH_3_), 2.59 (t, *J* = 6.8 Hz, 2H, COCH_2_), 2.86–2.87 (m, *J* = 6.0 Hz, 2H, CH_2_CH_3_), 4.04 (s, 2H, CH_2_Ph), 7.04–7.10 (m, 2H, H-6 and H-8), 7.18–7.25 (m, 5H, CH_2_PhH), 7.61 (d, *J* = 6.0 Hz, 1H, H-5). ^13^C-NMR (CDCl_3_): δ 18.17, 18.70, 27.21, 27.26, 31.85, 37.56, 39.06, 115.40, 122.14, 123.06, 128.81, 130.32, 131.37, 133.18, 133.57, 143.93, 157.39, 157.51, 158.47, 166.92, 176.71. HRMS (ESI): *m/z* 365.17447 (M+H^+^), 387.15629 (M+Na^+^), 403.13036 (M+K^+^).

*3-Benzyl-2-oxo-4-propyl-2H-chromen-7-yl pentanoate* (**51**). Yield: 0.166 g, 88%, white solid, m.p.: 78–79 °C. ^1^H-NMR (CDCl_3_): δ 0.95–1.05 (m, 6H, CH_3_), 1.43–1.53 (m, 4H, CH_2_CH_2_CH_2_CH_3_ and CH_2_CH_2_CH_3_), 1.74–1.79 (m, 2H, CH_2_CH_2_CH_2_CH_3_), 2.57–2.61 (m, 2H, CH_2_CH_2_CH_3_), 2.78–2.82 (m, 2H, COCH_2_), 4.04 (s, 2H, CH_2_Ph), 7.03–7.09 (m, 2H, H-6 and H-8), 7.18–7.25 (m, 5H, CH_2_PhH), 7.57–7.59 (m, 1H, H-5). ^13^C-NMR (CDCl_3_): δ 13.71, 14.48, 22.22, 22.47, 26.86, 31.15, 32.75, 34.07, 110.36, 117.47, 118.03, 124.19, 125.44, 126.40, 128.18, 128.57, 138.98, 151.20, 152.37, 153.39, 161.89, 171.73. HRMS (ESI): *m/z* 379.19074 (M+H^+^), 401.17263 (M+Na^+^), 417.14655 (M+K^+^).

*4-Ethyl-3-(4-fluorobenzyl)-2-oxo-2H-chromen-7-yl pentanoate* (**52**). Yield: 0.153 g, 80%, white solid, m.p.: 70–71 °C. ^1^H-NMR (CDCl_3_): δ 0.98 (t, *J* = 7.6 Hz, 3H, CH_2_CH_2_CH_2_CH_3_), 1.16 (t, *J* = 7.6 Hz, 3H, CH_2_CH_3_), 1.43–1.49 (m, *J* = 7.6 Hz, 2H, CH_2_CH_2_CH_2_CH_3_), 1.72–1.80 (m, *J* = 7.6 Hz, 2H, CH_2_CH_2_CH_2_CH_3_), 2.60 (t, *J* = 7.6 Hz, 2H, COCH_2_), 2.87 (q, *J* = 7.6 Hz, 2H, CH_2_CH_3_), 4.00 (s, 2H, CH_2_Ph), 6.95 (t, *J* = 8.4 Hz, 2H, ArH), 7.06 (d, *J* = 8.8 Hz, 1H, H-6), 7.11 (s, 1H, H-8), 7.20–7.23 (m, 2H, ArH), 7.62 (d, *J* = 8.4 Hz, 1H, H-5). ^13^C-NMR (CDCl_3_): δ 13.27, 13.70, 22.22, 22.26, 26.86, 31.82, 34.06, 110.46, 115.25, 115.46, 117.04, 118.17, 123.65, 125.37, 129.60, 129.67, 134.54, 134.57, 152.49, 152.57, 153.47, 160.34, 161.85, 162.76, 171.72. HRMS (ESI): *m/z* 383.16509 (M+H^+^), 405.14697 (M+Na^+^).

*3-(4-Fluorobenzyl)-4-methyl-2-oxo-2H-chromen-7-yl pentanoate* (**53**). Yield: 0.122 g, 66%, white solid, m.p.: 83–84 °C. ^1^H-NMR (CDCl_3_): δ 0.98 (t, *J* = 7.2 Hz, 3H, CH_2_CH_3_), 1.43–1.48 (m, *J* = 7.2 Hz, 2H, CH_2_CH_2_CH_2_CH_3_), 1.72–1.79 (m, *J* = 7.2 Hz, 2H, CH_2_CH_2_CH_2_CH_3_), 2.44 (s, 3H, CH_3_), 2.59 (t, *J* = 7.2 Hz, 2H, CH_2_CH_2_CH_2_CH_3_), 4.02 (s, 2H, CH_2_Ph), 6.95 (t, *J* = 8.0 Hz, 2H, ArH), 7.05–7.09 (m, 2H, H-6 and H-8), 7.20–7.26 (m, 2H, ArH), 7.62 (d, *J* = 8.8 Hz, 1H, H-5). ^13^C-NMR (CDCl_3_): δ 13.70, 15.47, 22.22, 26.85, 32.22, 34.06, 110.17, 115.26, 115.47, 118.12, 118.26, 124.62, 125.43, 129.67, 129.75, 134.33, 134.36, 146.98, 152.59, 152.90, 160.32, 161.54, 162.75, 171.69. HRMS (ESI): *m/z* 369.15012 (M+H^+^), 391.13209 (M+Na^+^), 407.10611 (M+K^+^).

*3-(4-Fluorobenzyl)-2-oxo-4-propyl-2H-chromen-7-yl pentanoate* (**54**). Yield: 0.122 g, 62%, white solid, m.p.: 68–70 °C. ^1^H-NMR (CDCl_3_): δ 0.98 (t, *J* = 7.2 Hz, 3H, CH_2_CH_2_CH_2_CH_3_), 1.05 (t, *J* = 7.2 Hz, 3H, CH_2_CH_2_CH_3_), 1.43–1.56 (m, 4H, CH_2_CH_2_CH_2_CH_3_ and CH_2_CH_2_CH_3_), 1.72–1.79 (m, *J* = 7.2 Hz, 2H, CH_2_CH_2_CH_2_CH_3_), 2.60 (t, *J* = 7.2 Hz, 2H, COCH_2_), 2.78–2.82 (m, *J* = 7.6 Hz, 2H, CH_2_CH_2_CH_3_), 4.00 (s, 2H, CH_2_Ph), 6.94–6.98 (m, 2H, CH_2_PhH), 7.05 (d, *J* = 8.8Hz, 1H, H-6), 7.10 (s, 1H, H-8), 7.20–7.23 (m, 2H, CH_2_PhH), 7.60 (d, *J* = 8.4 Hz, 1H, H-5). ^13^C-NMR (CDCl_3_): δ 13.70, 14.48, 22.22, 22.53, 26.86, 31.14, 32.01, 34.07, 110.41, 115.24, 115.45, 117.36, 118.11, 124.02, 125.48, 129.58, 129.66, 134.58, 134.61, 151.22, 152.46, 153.38, 160.34, 161.81, 162.77, 171.71. HRMS (ESI): *m/z* 397.18122 (M+H^+^), 419.16309 (M+Na^+^).

*3-Benzyl-4-methyl-2-oxo-2H-chromen-7-yl pivalate* (**55**). Yield: 0.157 g, 90%, white solid, m.p.: 157–158 °C. ^1^H-NMR (CDCl_3_): δ 1.37 (s, 9H, C(CH_3_)_3_), 2.44 (s, 3H, CH_3_), 4.06 (s, 2H, CH_2_Ph), 7.01 (d, *J* = 8.8 Hz, 1H, H-6), 7.06 (s, 1H, H-8), 7.19–7.26 (m, 5H, CH_2_Ph), 7.61 (d, *J* = 8.8 Hz, 1H, H-5). ^13^C-NMR (CDCl_3_): δ 15.51, 27.08, 32.99, 39.24, 110.06, 117.98, 118.29, 124.72, 125.33, 126.38, 128.28, 128.60, 138.75, 147.00, 152.94, 161.63, 176.52. HRMS (ESI): *m/z* 351.15890 (M+H^+^), 373.14087 (M+Na^+^), 389.11450 (M+K^+^).

*3-Benzyl-6-chloro-4-methyl-2-oxo-2H-chromen-7-yl pivalate* (**56**). Yield: 0.1 g, 87%, white solid, m.p.: 113 °C. ^1^H-NMR (CDCl_3_): δ 1.41 (s, 9H, C(CH_3_)_3_), 2.43 (s, 3H, CH_3_), 4.06 (s, 2H, CH_2_Ph), 7.11 (s, 1H, H-8), 7.20–7.28 (m, 5H, CH_2_PhH), 7.66 (s, 1H, H-5). ^13^C-NMR (CDCl_3_): δ 15.55, 27.14, 33.08, 39.43, 112.26, 119.52, 123.06, 125.64, 125.94, 126.51, 128.27, 128.66, 138.38, 145.92, 148.81, 151.19, 161.06, 175.56. HRMS (ESI): *m/z* 385.12023 (M+H^+^), 407.10224 (M+Na^+^), 423.07644 (M+K^+^).

*3-Benzyl-4-ethyl-2-oxo-2H-chromen-7-yl pivalate* (**57**). Yield: 0.180 g, 99%, white solid, m.p.: 105–106 °C. ^1^H-NMR (CDCl_3_): δ 1.15 (t, *J* = 7.6 Hz, 3H, CH_2_CH_3_), 1.38 (s, 9H, C(CH_3_)_3_), 2.87 (q, *J* = 7.6 Hz, 2H, CH_2_CH_3_), 4.05 (s, 2H, CH_2_Ph), 7.01–7.03 (dd, *J* = 1.6, 8.4 Hz, 1H, H-6), 7.07 (d, *J* = 2.0 Hz, 1H, H-8), 7.19–7.30 (m, 5H, CH_2_Ph), 7.61 (d, *J* = 8.8 Hz, 1H, H-5). ^13^C-NMR (CDCl_3_): δ 13.23, 22.29, 27.08, 32.57, 39.25, 110.34, 117.06, 118.04, 123.76, 125.30, 126.39, 128.21, 128.59, 138.95, 152.57, 152.83, 153.50, 161.97, 176.58. HRMS (ESI): *m/z* 365.17453 (M+H^+^), 387.15631 (M+Na^+^), 403.13040 (M+K^+^).

*3-Benzyl-2-oxo-4-propyl-2H-chromen-7-yl pivalate* (**58**). Yield: 0.184 g, 97%, white solid, m.p.: 138–139 °C. ^1^H-NMR (CDCl_3_): δ 1.04 (t, *J* = 6.4 Hz, 3H, CH_2_CH_3_), 1.38 (s, 9H, C(CH_3_)_3_), 1.52 (q, *J* = 7.2 Hz, 2H, CH_2_CH_2_CH_3_), 2.81 (m, 2H, CH_2_CH_2_CH_3_), 4.04 (s, 2H, CH_2_Ph), 7.01 (d, *J* = 8.4 Hz, 1H, H-6), 7.07 (s, 1H, H-8), 7.19–7.26 (m, 5H, CH_2_Ph), 7.58 (d, *J* = 8.4 Hz, 1H, H-5). ^13^C-NMR (CDCl_3_): δ 14.48, 22.48, 27.08, 31.15, 32.77, 39.25, 110.28, 117.39, 117.96, 124.14, 125.39, 126.39, 128.20, 128.57, 139.00, 151.21, 152.82, 153.42, 161.90, 176.54. HRMS (ESI): *m/z* 379.19072 (M+H^+^), 401.17258 (M+Na^+^), 417.14652 (M+K^+^).

*4-Ethyl-3-(4-fluorobenzyl)-2-oxo-2H-chromen-7-yl pivalate* (**59**). Yield: 0.16 g, 84%, white solid, m.p.: 103–104 °C. ^1^H-NMR (CDCl_3_): δ 1.16 (t, *J* = 7.6 Hz, 3H, CH_2_CH_3_), 1.38 (s, 9H, C(CH_3_)_3_), 2.87 (q, *J* = 7.6 Hz, 2H, CH_2_CH_3_), 4.00 (s, 2H, CH_2_Ph), 6.95 (t, *J* = 8.4 Hz, 2H, ArH), 7.03 (d, *J* = 8.8 Hz, 1H, H-6), 7.08 (s, 1H, H-8), 7.21–7.26 (m, 2H, ArH), 7.62 (d, *J* = 8.4 Hz, 1H, H-5). ^13^C-NMR (CDCl_3_): δ 13.28, 22.27, 27.07, 31.83, 39.25, 110.37, 115.24, 115.45, 116.95, 118.11, 123.59, 125.34, 129.62, 129.69, 134.57, 134.60, 152.58, 152.94, 153.50, 160.33, 161.86, 162.76, 176.53. HRMS (ESI): *m/z* 383.16508 (M+H^+^), 405.14694 (M+Na^+^), 421.11022 (M+K^+^).

*3-(4-Fluorobenzyl)-4-methyl-2-oxo-2H-chromen-7-yl pivalate* (**60**). Yield: 0.096 g, 52%, white solid, m.p.: 136–138 °C. ^1^H-NMR (CDCl_3_): δ 1.38 (s, 9H, C(CH_3_)_3_), 2.45 (s, 3H, CH_3_), 4.02 (s, 2H, CH_2_Ph), 6.96 (t, *J* = 8.0 Hz, 2H, ArH), 7.01–7.06 (m, 2H, H-6 and H-8), 7.21–7.26 (m, 2H, ArH), 7.60 (d, *J* = 8.8 Hz, 1H, H-5). ^13^C-NMR (CDCl_3_): δ 15.48, 27.07, 32.24, 39.25, 110.11, 115.27, 115.48, 118.06, 118.19, 124.59, 125.37, 129.68, 129.76, 134.35, 134.38, 146.98, 152.94, 153.04, 160.33, 161.55, 162.76, 176.52. HRMS (ESI): *m/z* 369.15012 (M+H^+^), 391.13212 (M+Na^+^), 407.10589 (M+K^+^).

*3-(4-Fluorobenzyl)-2-oxo-4-propyl-2H-chromen-7-yl pivalate* (**61**). Yield: 0.160 g, 81%, white solid, m.p.: 138–140 °C. ^1^H-NMR (CDCl_3_): δ 1.05 (t, *J* = 7.2 Hz, 3H, CH_2_CH_3_), 1.38 (s, 9H, C(CH_3_)_3_), 1.52–1.56 (m, *J* = 7.2 Hz, 2H, CH_2_CH_2_CH_3_), 2.81 (t, *J* = 7.6 Hz, 2H, CH_2_CH_2_CH_3_), 4.00 (s, 2H, CH_2_Ph), 6.96 (t, *J* = 8.4 Hz, 2H, ArH), 7.02 (d, *J* =8.8 Hz, 1H, H-6), 7.07 (s, 1H, H-8), 7.20–7.23 (m, 2H, ArH), 7.59 (d, *J* = 8.8 Hz, 1H, H-5). ^13^C-NMR (CDCl_3_): δ 14.48, 22.53, 27.07, 31.14, 32.02, 39.25, 110.33, 115.24, 115.45, 117.28, 118.05, 123.98, 125.42, 129.59, 129.67, 134.60, 134.63, 151.22, 152.91, 153.41, 160.34, 161.82, 162.77, 176.53. HRMS (ESI): *m/z* 397.18136 (M+H^+^), 419.16318 (M+Na^+^).

*3-Benzyl-4-methyl-2-oxo-2H-chromen-7-yl benzoate* (**62**). Yield: 0.180 g, 97%, white solid, m.p.: 149–150 °C. ^1^H-NMR (CDCl_3_): δ 2.47 (s, 3H, CH_3_), 4.08 (s, 2H, CH_2_Ph), 7.18–7.27 (m, 7H, ArH), 7.53 (t, 2H, ArH), 7.67 (t, 2H, ArH), 8.21 (d, *J* = 7.6 Hz, 2H, ArH). ^13^C-NMR (CDCl_3_): δ 15.55, 33.01, 110.33, 118.18, 118.55, 124.88, 125.49, 126.41, 128.29, 128.62, 128.72, 128.91, 130.33, 134.02, 138.72, 147.03, 152.69, 152.99, 161.64, 164.62. HRMS (ESI): *m/z* 371.12762 (M+H^+^), 393.10954 (M+Na^+^), 409.08341 (M+K^+^).

*3-Benzyl-6-chloro-4-methyl-2-oxo-2H-chromen-7-yl benzoate* (**63**). Yield: 0.179 g, 88.6%, white solid, m.p.: 133–135 °C. ^1^H-NMR (CDCl_3_): δ 2.46 (s, 3H, CH_3_), 4.08 (s, 2H, CH_2_Ph), 7.21–7.32 (m, 6H, ArH), 7.53–7.57 (m, 2H, ArH), 7.67–7.72 (m, 2H, ArH), 8.23–8.25 (m, *J* = 7.2 Hz, 2H, ArH). ^13^C-NMR (CDCl_3_): δ 15.59, 33.10, 112.47, 119.71, 123.11, 125.71, 126.08, 126.54, 128.27, 128.68, 128.78, 130.53, 134.26, 138.36, 145.94, 148.67, 151.21, 161.08, 163.77. HRMS (ESI): *m/z* 405.08872 (M+H^+^), 427.07096 (M+Na^+^), 443.04450 (M+K^+^).

*3-Benzyl-4-ethyl-2-oxo-2H-chromen-7-yl benzoate* (**64**). Yield: 0.149 g, 78%, white solid, m.p.: 138 °C. ^1^H-NMR (CDCl_3_): δ 1.17 (t, *J* = 6.8 Hz, 3H, CH_2_CH_3_), 2.88–2.90 (m, *J* = 7.2 Hz, 2H, CH_2_CH_3_), 4.06 (s, 2H, CH_2_Ph), 7.18–7.27 (m, 7H, ArH), 7.52–7.55 (m, 2H, ArH), 7.65–7.69 (m, 2H, ArH), 8.21 (d, *J* = 6.4 Hz, 2H, ArH). ^13^C-NMR (CDCl_3_): δ 18.20, 27.29, 37.60, 115.58, 122.32, 123.18, 128.94, 130.41, 131.38, 133.21, 133.58, 133.69, 133.94, 135.31, 138.99, 143.94, 157.50, 157.60, 158.57, 166.90, 169.60. HRMS (ESI): *m/z* 385.14322 (M+H^+^), 407.12515 (M+Na^+^), 423.09884 (M+K^+^).

*3-Benzyl-2-oxo-4-propyl-2H-chromen-7-yl benzoate* (**65**). Yield: 0.159 g, 80.3%, white solid, m.p.: 120 °C. ^1^H-NMR (CDCl_3_): δ 1.05 (t, *J* = 7.2 Hz, 3H, CH_2_CH_3_), 1.52–1.57 (m, *J* = 7.2 Hz, 2H, CH_2_CH_3_), 2.83 (t, *J* = 7.6 Hz, 2H, CH_2_CH_2_CH_3_), 4.06 (s, 2H, CH_2_Ph), 7.18–7.27 (m, 7H, ArH), 7.48–7.55 (m, 2H, ArH), 7.64–7.68 (m, 2H, ArH), 8.21 (d, *J* = 7.2 Hz, 2H, ArH). ^13^C-NMR (CDCl_3_): δ 14.51, 22.50, 31.19, 32.79, 110.56, 117.66, 118.18, 124.30, 125.56, 126.42, 128.21, 128.60, 128.72, 130.34, 134.03, 138.98, 151.24, 152.57, 153.46, 161.92, 164.64. HRMS (ESI): *m/z* 399.15917 (M+H^+^), 421.14128 (M+Na^+^), 437.11514 (M+K^+^).

*3-(4-Fluorobenzyl)-4-methyl-2-oxo-2H-chromen-7-yl benzoate* (**66**). Yield: 0.13 g, 67%, white solid, m.p.: 151–153 °C. ^1^H-NMR (CDCl_3_): δ 2.48 (s, 3H, CH_3_), 4.04 (s, 2H, CH_2_Ph), 6.97 (t, *J* = 8.4 Hz, 2H, ArH), 7.20–7.26 (m, 4H, ArH), 7.52–7.56 (m, 2H, ArH), 7.65–7.69 (m, 2H, ArH), 8.21 (d, *J* = 7.6 Hz, 2H, ArH). ^13^C-NMR (CDCl_3_): δ 15.51, 32.25, 110.39, 115.29, 115.50, 118.26, 118.44, 124.75, 125.53, 128.72, 128.87, 129.69, 129.77, 130.33, 134.04, 134.35, 146.99, 152.79, 152.99, 160.35, 161.56, 162.78,164.61. HRMS (ESI): *m/z* 389.11849 (M+H^+^), 411.10049 (M+Na^+^), 427.07456 (M+K^+^).

*4-Ethyl-3-(4-fluorobenzyl)-2-oxo-2H-chromen-7-yl benzoate* (**67**). Yield: 0.073 g, 36%, white solid, m.p.: 70–71 °C. ^1^H-NMR (CDCl_3_): δ 1.18 (t, *J* = 7.2 Hz, 3H, CH_2_CH_3_), 2.89 (q, *J* = 7.2 Hz, 2H, CH_2_CH_3_), 4.02 (s, 2H, CH_2_Ph), 6.96 (t, *J* = 8.0 Hz, 2H, ArH), 7.22–7.25 (m, 4H, ArH), 7.53–7.69 (m, 4H, ArH), 8.21 (d, *J* = 7.2 Hz, 2H, ArH). ^13^C-NMR (CDCl_3_): δ 18.27, 27.27, 36.85, 115.62, 120.24, 120.46, 122.21, 123.27, 128.77, 130.45, 133.70, 133.90, 134.61, 134.69, 135.31, 139.01, 139.54, 157.53, 157.70, 158.55, 166.82, 167.78, 169.59. HRMS (ESI): *m/z* 403.13421 (M+H^+^).

*3-(4-Fluorobenzyl)-2-oxo-4-propyl-2H-chromen-7-yl benzoate* (**68**). Yield: 0.176 g, 85%, white solid, m.p.: 95–96 °C. ^1^H-NMR (CDCl_3_): δ 1.06 (t, *J* = 7.2 Hz, 3H, CH_2_CH_3_), 1.53–1.58 (m, *J* = 7.2 Hz, 2H, CH_2_CH_2_CH_3_), 2.83 (t, *J* = 7.6 Hz, 2H, CH_2_CH_2_CH_3_), 4.02 (s, 2H, CH_2_Ph), 6.96 (t, *J* = 8.4 Hz, 2H, ArH), 7.19–7.25 (m, 4H, ArH), 7.52–7.55 (m, 2H, ArH), 7.65–7.69 (m, 2H, ArH), 8.21 (d, *J* = 8.0 Hz, 2H, ArH). ^13^C-NMR (CDCl_3_): δ 14.51, 22.56, 31.18, 32.04, 110.60, 115.26, 115.47, 117.54, 118.26, 124.13, 125.60, 128.73, 128.87, 129.61, 129.68, 130.33, 134.05, 134.59, 134.62, 151.26, 152.66, 153.45, 160.35, 161.83, 162.78, 164.62. HRMS (ESI): *m/z* 417.14989 (M+H^+^), 439.13191 (M+Na^+^), 455.10528 (M+K^+^).

*3-Benzyl-4-methyl-2-oxo-2H-chromen-7-yl benzenesulfonate* (**69**). Yield: 0.138 g, 68%, white solid, m.p.: 131–132 °C. ^1^H-NMR (CDCl_3_): δ 2.43 (s, 3H, CH_3_), 4.04 (s, 2H, CH_2_Ph), 6.84 (s, 1H, H-8), 7.06 (d, *J* = 8.8 Hz, 1H, H-6), 7.19–7.29 (m, 5H, CH_2_Ph), 7.53–7.58 (m, 3H, H-5 and PhSO_3_), 7.70 (t, *J* = 7.6 Hz, 1H, PhSO_3_), 7.86 (d, *J* = 7.6 Hz, 2H, PhSO_3_). ^13^C-NMR (CDCl_3_): δ 15.54, 33.03, 110.61, 118.69, 119.57, 125.67, 125.80, 126.51, 128.31, 128.46, 128.64, 129.43, 134.70, 135.03, 138.45, 146.56, 150.76, 152.59, 161.20. HRMS (ESI): *m/z* 407.09446 (M+H^+^), 429.07635 (M+Na^+^), 445.05041 (M+K^+^).

*3-Benzyl-6-chloro-4-methyl-2-oxo-2H-chromen-7-yl benzenesulfonate* (**70**). Yield: 0.165 g, 75%, white solid, m.p.: 154–155 °C. ^1^H-NMR (CDCl_3_): δ 2.41 (s, 3H, CH_3_), 4.05 (s, 2H, CH_2_Ph), 7.19–7.25 (m, 6H, ArH), 7.58–7.60 (m, 3H, ArH), 7.73 (m, 1H, ArH), 7.93 (d, *J* = 6.4 Hz, 2H, ArH). ^13^C-NMR (CDCl_3_): δ 15.54, 33.13, 112.33, 120.38, 123.50, 126.11, 126.62, 126.78, 128.30, 128.60, 128.70, 129.47, 134.91, 135.37, 138.15, 145.50, 146.48, 150.83, 160.73. HRMS (ESI): *m/z* 441.05606 (M+H^+^), 463.03794 (M+Na^+^), 479.01170 (M+K^+^).

*3-Benzyl-4-ethyl-2-oxo-2H-chromen-7-yl benzenesulfonate* (**71**). Yield: 0.187 g, 89%, white solid, m.p.: 57–58 °C. ^1^H-NMR (CDCl_3_): δ 1.11 (t, *J* = 7.2 Hz, 3H, CH_2_CH_3_), 2.84 (t, *J* = 7.2 Hz, 2H, CH_2_CH_3_), 4.01 (s, 2H, CH_2_Ph), 6.88 (s, 1H, H-8), 7.03 (d, *J* = 8.8 Hz, 1H, H-6), 7.16–7.24 (m, 5H, CH_2_Ph), 7.52–7.58 (m, 3H, H-5 and PhSO_3_), 7.67–7.70 (m, *J* = 7.2 Hz, 1H, PhSO_3_), 7.86 (d, *J* = 8.0 Hz, 2H, PhSO_3_). ^13^C-NMR (CDCl_3_): δ 18.15, 27.28, 37.62, 115.83, 123.38, 123.66, 129.67, 130.88, 131.48, 133.24, 133.42, 133.61, 134.48, 139.76, 140.02, 143.69, 155.63, 157.23, 158.14, 166.51. HRMS (ESI): *m/z* 421.11015 (M+H^+^), 443.09190 (M+Na^+^), 459.06604 (M+K^+^).

*3-Benzyl-2-oxo-4-propyl-2H-chromen-7-yl benzenesulfonate* (**72**). Yield: 0.155 g, 71%, white solid, m.p.: 96 °C. ^1^H-NMR (CDCl_3_): δ 1.03 (br s, 3H, CH_2_CH_3_), 1.49 (br s, 2H, CH_2_CH_3_), 2.79 (br s, 2H, CH_2_CH_2_CH_3_), 4.02 (s, 2H, CH_2_Ph), 6.84 (s, 1H, H-8), 7.06 (d, *J* = 6.8 Hz, 1H, H-6), 7.24 (m, 5H, CH_2_Ph), 7.56 (br s, 3H, H-5 and PhSO_3_), 7.71 (br s, 1H, PhSO_3_), 7.87 (d, *J* = 6.0 Hz, 2H, PhSO_3_). ^13^C-NMR (CDCl_3_): δ 14.48, 22.43, 31.15, 32.81, 110.81, 118.71, 125.07, 125.86, 126.52, 128.21, 128.46, 128.62, 129.45, 134.71, 135.09, 138.69, 150.64, 150.80, 153.06, 161.48. HRMS (ESI): *m/z* 435.12627 (M+H^+^), 457.10839 (M+Na^+^), 473.08227 (M+K^+^).

*4-Ethyl-3-(4-fluorobenzyl)-2-oxo-2H-chromen-7-yl benzenesulfonate* (**73**). Yield: 0.195 g, 89%, white solid, m.p.: 93–94 °C. ^1^H-NMR (CDCl_3_): δ 1.14 (t, *J* = 7.6 Hz, 3H, CH_2_CH_3_), 2.85 (q, *J* = 7.6 Hz, 2H, CH_2_CH_3_), 3.97 (s, 2H, CH_2_Ph), 6.87 (s, 1H, H-8), 6.94 (t, *J* = 8.4 Hz, 2H, CH_2_Ph), 7.07 (d, *J* = 8.4 Hz, 1H, H-6), 7.19–7.23 (m, 2H, CH_2_Ph), 7.55–7.60 (m, 3H, H-5 and PhSO_3_), 7.69–7.73 (m, 1H, PhSO_3_), 7.87 (d, *J* = 8.0 Hz, 2H, PhSO_3_). ^13^C-NMR (CDCl_3_): δ 13.22, 22.26, 31.87, 110.89, 115.28, 115.49, 118.26, 118.79, 124.53, 125.83, 128.44, 129.45, 129.65, 129.73, 134.27, 134.30, 134.74, 135.08, 150.73, 152.19, 153.15, 160.36, 161.44, 162.79. HRMS (ESI): *m/z* 439.10053 (M+H^+^), 461.08240 (M+Na^+^), 477.05639 (M+K^+^).

*3-(4-Fluorobenzyl)-4-methyl-2-oxo-2H-chromen-7-yl benzenesulfonate* (**74**). Yield: 0.153 g, 72%, white solid, m.p.: 108–109 °C. ^1^H-NMR (CDCl_3_): δ 2.44 (s, 3H, CH_3_), 3.99 (s, 2H, CH_2_Ph), 6.85 (s, 1H, H-8), 6.93–6.97 (m, *J* = 8.4 Hz, 2H, CH_2_Ph), 7.07 (d, *J* = 8.8 Hz, 1H, H-6), 7.19–7.23 (m, 2H, CH_2_Ph), 7.54–7.59 (m, 3H, H-5 and PhSO_3_), 7.69–7.71 (m, 1H, PhSO_3_), 7.86 (d, *J* = 7.6 Hz, 2H, PhSO_3_). ^13^C-NMR (CDCl_3_): δ 15.50, 32.27, 110.65, 115.31, 115.52, 118.76, 119.47, 125.52, 125.86, 128.45, 129.44, 129.72, 129.80, 134.05, 134.09, 134.72, 135.03, 146.56, 150.84, 152.59, 160.36, 161.13, 162.80. HRMS (ESI): *m/z* 425.08559 (M+H^+^), 447.06760 (M+Na^+^), 463.04156 (M+K^+^).

*3-(4-Fluorobenzyl)-2-oxo-4-propyl-2H-chromen-7-yl benzenesulfonate* (**75**). Yield: 0.104 g, 46%, white solid, m.p.: 109–110 °C. ^1^H-NMR (CDCl_3_): δ 1.04 (t, *J* = 7.2 Hz, 3H, CH_2_CH_3_), 1.48–1.54 (m, *J* = 7.6 Hz, 2H, CH_2_CH_2_CH_3_), 2.79 (t, *J* = 7.6 Hz, 2H, CH_2_CH_2_CH_3_), 3.97 (s, 2H, CH_2_Ph), 6.86 (s, 1H, H-8), 6.95 (t, *J* = 8.4 Hz, 2H, CH_2_Ph), 7.07 (d, *J* = 8.8 Hz, 1H, H-6), 7.19–7.22 (m, 2H, CH_2_Ph), 7.55–7.59 (m, 3H, H-5 and PhSO_3_), 7.69–7.73 (m, 1H, PhSO_3_), 7.88 (d, *J* = 7.6 Hz, 2H, PhSO_3_). ^13^C-NMR (CDCl_3_): δ 14.48, 22.48, 31.13, 32.05, 110.85, 115.29, 115.50, 118.56, 118.76, 124.89, 125.90, 128.45, 129.45, 129.63, 129.70, 134.32, 134.72, 135.10, 150.72, 150.82, 153.07, 160.37, 161.40, 162.81. HRMS (ESI): *m/z* 453.11683 (M+H^+^), 475.09903 (M+Na^+^), 491.07253 (M+K^+^).

*3-Benzyl-4-methyl-2-oxo-2H-chromen-7-yl 4-methylbenzenesulfonate* (**76**). Yield: 0.103 g, 49%, white solid, m.p.: 138–139 °C. ^1^H-NMR (CDCl_3_): δ 2.43 (s, 3H, CH_3_), 2.46 (s, 3H, CH_3_), 4.03 (s, 2H, CH_2_Ph), 6.82 (s, 1H, H-8), 7.08 (d, *J* = 8.8 Hz, 1H, H-6), 7.18–7.28 (m, 5H, CH_2_Ph), 7.33 (d, *J* = 8.0 Hz, 2H, PhSO_3_), 7.57 (d, *J* = 8.8 Hz, 1H, H-5), 7.72 (d, *J* = 8.0 Hz, 2H, PhSO_3_). ^13^C-NMR (CDCl_3_): δ 15.54, 21.79, 33.02, 110.59, 118.80, 119.49, 125.57, 125.79, 126.50, 128.31, 128.48, 128.63, 130.07, 131.96, 138.48, 146.00, 146.65, 150.88, 152.55, 161.26. HRMS (ESI): *m/z* 421.11003 (M+H^+^), 443.09190 (M+Na^+^), 459.06602 (M+K^+^).

*3-Benzyl-6-chloro-4-methyl-2-oxo-2H-chromen-7-yl 4-methylbenzenesulfonate* (**77**). Yield: 0.136 g, 60%, white solid, m.p.: 173–174 °C. ^1^H-NMR (CDCl_3_): δ 2.41 (s, 3H, CH_3_), 2.48 (s, 3H, CH_3_), 4.04 (s, 2H, CH_2_Ph), 7.16 (s, 1H, H-8), 7.19–7.27 (m, 5H, CH_2_PhH), 7.36 (d, *J* = 8.0 Hz, 2H, ArH), 7.60 (s, 1H, H-5), 7.80 (d, *J* = 7.6 Hz, 2H, ArH). ^13^C-NMR (CDCl_3_): δ 15.54, 21.83, 33.11, 112.23, 120.28, 123.60, 126.10, 126.61, 126.68, 128.30, 128.63, 128.69, 130.10, 132.32, 138.17, 145.57, 146.26, 146.61, 150.81, 160.78. HRMS (ESI): *m/z* 455.07186 (M+H^+^).

*3-Benzyl-4-ethyl-2-oxo-2H-chromen-7-yl 4-methylbenzenesulfonate* (**78**). Yield: 0.169 g, 78%, white solid, m.p.: 66–68 °C. ^1^H-NMR (CDCl_3_): δ 1.13 (t, *J* = 7.6 Hz, 3H, CH_2_CH_3_), 2.46 (s, 3H, CH_3_), 2.85 (q, *J* = 7.6 Hz, 2H, CH_2_CH_3_), 4.01 (s, 2H, CH_2_Ph), 6.84 (d, *J* = 1.6 Hz, 1H, H-8), 7.07–7.10 (dd, *J* = 1.6, 8.8 Hz, 1H, H-6), 7.18–7.26 (m, 5H, CH_2_Ph), 7.34 (d, *J* = 8.0 Hz, 2H, PhSO_3_), 7.57 (d, *J* = 8.8 Hz, 1H, H-5), 7.74 (d, *J* = 8.0 Hz, 2H, PhSO_3_). ^13^C-NMR (CDCl_3_): δ 18.14, 26.77, 27.26, 37.60, 115.83, 123.27, 123.83, 129.61, 130.74, 131.48, 133.22, 133.46, 133.60, 135.06, 137.05, 143.67, 150.98, 155.77, 157.18, 158.12, 166.55. HRMS (ESI): *m/z* 435.12593 (M+H^+^), 457.10761 (M+Na^+^), 473.08160 (M+K^+^).

*3-Benzyl-2-oxo-4-propyl-2H-chromen-7-yl 4-methylbenzenesulfonate* (**79**). Yield: 0.159 g, 80%, white solid, m.p.: 120 °C. ^1^H-NMR (CDCl_3_): δ 1.04 (t, *J* = 7.2 Hz, 3H, CH_2_CH_3_), 1.48 (q, *J* = 7.2 Hz, 3H, CH_2_CH_3_), 2.47 (s, 3H, CH_3_), 2.79 (q, *J* = 7.6 Hz, 2H, CH_2_CH_2_CH_3_), 4.02 (s, 2H, CH_2_Ph), 6.81 (s, 1H, H-8), 7.09 (d, *J* = 8.4 Hz, 1H, H-6), 7.19–7.25 (m, 5H, CH_2_Ph), 7.34 (d, *J* = 7.6 Hz, 2H, PhSO_3_), 7.55 (d, *J* = 8.8 Hz, 1H, H-5), 7.74 (d, *J* = 7.6 Hz, 2H, PhSO_3_). ^13^C-NMR (CDCl_3_): δ 14.48, 21.80, 22.44, 31.15, 32.80, 110.80, 118.59, 118.84, 124.98, 125.83, 126.51, 128.21, 128.48, 128.61, 130.07, 132.04, 138.71, 145.99, 150.76, 150.86, 153.03, 161.54. HRMS (ESI): *m/z* 449.14190 (M+H^+^), 471.12381 (M+Na^+^), 487.09789 (M+K^+^).

*4-Ethyl-3-(4-fluorobenzyl)-2-oxo-2H-chromen-7-yl 4-methylbenzenesulfonate* (**80**). Yield: 0.132 g, 58%, white solid, m.p.: 96–97 °C. ^1^H-NMR (CDCl_3_): δ 1.15 (t, *J* = 7.2 Hz, 3H, CH_2_CH_3_), 2.47 (s, 2H, CH_3_), 2.85 (q, *J* = 7.2 Hz, 2H, CH_2_CH_3_), 3.97 (s, 2H, CH_2_Ph), 6.84 (s, 1H, H-8), 6.95 (t, *J* = 8.0 Hz, 2H, CH_2_Ph), 7.10 (d, *J* = 8.4 Hz, 1H, H-6), 7.21 (m, 2H, CH_2_Ph), 7.35 (t, *J* = 7.6 Hz, 2H, PhSO_3_), 7.59 (d, *J* = 8.8 Hz, 1H, H-5), 7.74 (d, *J* = 7.6 Hz, 2H, PhSO_3_). ^13^C-NMR (CDCl_3_): δ 13.22, 21.80, 22.27, 31.86, 110.89, 115.29, 115.50, 118.17, 118.96, 124.45, 125.77, 128.47, 129.64, 129.72, 130.08, 132.04, 134.27, 134.30, 146.02, 150.85, 152.23, 153.12, 160.37, 161.50, 162.80. HRMS (ESI): *m/z* 453.11738 (M+H^+^).

*3-(4-Fluorobenzyl)-4-methyl-2-oxo-2H-chromen-7-yl 4-methylbenzenesulfonate* (**81**). Yield: 0.145 g, 66%, white solid, m.p.: 95–96 °C. ^1^H-NMR (CDCl_3_): δ 2.44 (s, 3H, CH_3_), 2.47(s, 3H, CH_3_), 3.99 (s, 2H, CH_2_Ph), 6.83 (s, 1H, H-8), 6.93–6.97 (m, *J* = 8.4 Hz, 2H, CH_2_Ph), 7.09 (d, *J* = 8.8 Hz, 1H, H-6), 7.20–7.22 (m, 2H, CH_2_Ph), 7.34 (d, *J* = 7.6 Hz, 2H, PhSO_3_), 7.58 (d, *J* = 8.4 Hz, 1H, H-5), 7.72 (d, *J* = 8.0 Hz, 2H, PhSO_3_). ^13^C-NMR (CDCl_3_): δ 15.51, 21.79, 32.26, 110.64, 115.31, 115.52, 118.88, 119.38, 125.42, 125.83, 128.47, 129.72, 129.80, 130.07, 131.98, 134.08, 134.11, 146.02, 146.63, 150.95, 152.56, 160.36, 161.19, 162.79. HRMS (ESI): *m/z* 439.10127 (M+H^+^), 461.08292 (M+Na^+^), 477.05669 (M+K^+^).

*3-(4-Fluorobenzyl)-2-oxo-4-propyl-2H-chromen-7-yl 4-methylbenzenesulfonate* (**82**). Yield: 0.134 g, 58%, white solid, m.p.: 110–111 °C. ^1^H-NMR (CDCl_3_): δ 1.05 (t, *J* = 7.2 Hz, 3H, CH_2_CH_3_), 1.49–1.54 (m, *J* = 7.2 Hz, 2H, CH_2_CH_2_CH_3_), 2.47 (s, 3H, CH_3_), 2.79 (t, *J* = 7.6 Hz, 2H, CH_2_CH_2_CH_3_), 3.98 (s, 2H, CH_2_Ph), 6.83 (s, 1H, H-8), 6.93–6.97 (m, *J* = 6.8 Hz, 2H, CH_2_Ph), 7.10 (d, *J* = 8.8 Hz, 1H, H-6), 7.19–7.22 (m, 2H, CH_2_Ph), 7.35 (d, *J* = 7.2 Hz, 2H, PhSO_3_), 7.56 (d, *J* = 8.8 Hz, 1H, H-5), 7.74 (d, *J* = 6.8 Hz, 2H, PhSO_3_). ^13^C-NMR (CDCl_3_): δ 14.48, 21.80, 22.49, 31.14, 32.05, 110.84, 115.28, 115.50, 118.48, 118.90, 124.80, 125.87, 128.47, 129.62, 129.70, 130.07, 132.05, 134.31, 134.34, 146.01, 150.84, 150.89, 153.03, 160.37, 161.46, 162.80. HRMS (ESI): *m/z* 467.13229 (M+H^+^), 489.11399 (M+Na^+^), 505.08809 (M+K^+^).

#### 3.2.6. Procedure for the Preparation of 3-Benzyl-7-methoxy-2H-chromen-2-one (**83**) [25]

Step 1: Preparation of 2-hydroxy-4-methoxybenzaldehyde (**6**) [35]

To the solution of compound 2,4-dimethoxybenzaldehyde (5, 10 g, 60 mmol, 1 equiv.) in dry dichloromethane (200 mL) was added dropwise AlCl_3_ (20 g. 150 mmol, 2.5 equiv.) at room temperature, the mixture was stirred for 1 h and poured into 3 mol/L HCl solution. The reaction mixture was extractrd with CH_2_Cl_2_ three times and the organic extracts were combined, washed with brine and dried over MgSO_4_. The solvent was removed under reduced pressure. Purification of the residue by flash column chromatography (PE-EA = 20:1) to afford 2-hydroxy-4-methoxybenzaldehyde (6) as a white powder (7.917 g, 86.7%), m.p.: 35–36 °C. ^1^H-NMR (CDCl_3_): δ 3.86 (s, 3H, CH_3_), 6.43 (d, *J* = 2.4 Hz, 1H, PhH), 6.53–6.56 (dd, *J* = 2.4, 8.8 Hz, 1H, PhH), 7.43 (d, *J* = 8.8 Hz, 1H, PhH), 9.72 (s, 1H, OH), 11.49 (s, 1H, CHO).

Step 2: Preparation of ethoxycarbonylbenzylmethylene triphenylphosphorane (**8a**)

To a solution of benzyl bromide (0.3 mL, 2.53 mmol, 1.05 equiv.) in dry chloroform (20 mL), ethoxycarbonyl methylene triphenylphosphorane (**7**, 0.835 g, 2.4 mmol, 1 equiv.) was added at room temperature. After stirring for 30 min at r.t., the mixture was refluxed for 15 h. The solvent was removed under reduced pressure to afford the phosphonium salt as an oil. This salt was dissolved in water (50 mL) and dichloromethane (50 mL) was added. It was then made alkaline (phenolphthalein colorless to pink) with 2 M NaOH. The dichloromethane layer was separated and the aqueous layer was extracted with dichloromethane (50 mL × 2). The combined dichloromethane extract was dried over MgSO_4_ and solvent was removed to give the phosphorane, which was recrystallised from chloroform-hexane as yellowish needles (0.680 g, 81.3%), m.p.: 133–134 °C. MS (ESI): *m/z* 439.2 (M+H^+^).

Step 3: Preparation of (*E*)-ethyl 2-benzyl-3-(2-hydroxy-4-methoxyphenyl) acrylate (**9a**)

A mixture of compound **6** (0.858 g, 5.645 mmol, 1 equiv.) and compound **8a** (3.045 g, 6.944 mmol, 1.23 equiv.) was refluxed at 85 °C in dry benzene (30 mL) for 15 h. The solvent was removed under reduced pressure. The residue obtained was purified by flash column chromatography (PE-EA = 5:1) to afford the crude product, which was recrystallised from chloroform-hexane as a white solid (1.233 g, 70%), m.p.:104–105 °C. ^1^H-NMR (CDCl_3_): δ 1.22 (t, *J* = 7.2 Hz, 3H, CH_2_CH_3_), 3.77 (s, 3H, OCH_3_), 3.87 (s, 2H, CH_2_Ph), 4.19 (q, *J* = 7.2 Hz, 2H, CH_2_CH_3_), 5.34–5.65 (br s, 1H, OH), 6.41–6.44 (m, 2H, PhH), 7.12–7.28 (m, 6H, PhH), 7.94 (s, 1H, CH=C). MS (ESI): *m/z* 313.1 (M+H^+^), 330.1 (M+H_2_O), 335.1 (M+Na^+^), 351.1 (M+K^+^), 647.2 (2M+Na^+^).

Step 4: Preparation of 3-benzyl-7-methoxy-2*H*-chromen-2-one (**83**)

Compound **9a** (6 mmol, 1.884 g) was heated at 220 °C for 2 h under nitrogen atmosphere, The residue obtained was purified by flash column chromatography (PE-EA = 10:1) to afford the product, a white to light green powder (1.449 g, 90%), m.p.: 102–103 °C. ^1^H-NMR (CDCl_3_): δ 3.85 (s, 5H, OCH_3_, CH_2_Ph), 6.77–6.81 (m, 2H, PhH), 7.22–7.36 (m, 7H, PhH). MS (ESI): *m/z* 267.1 (M+H^+^), 289.1 (M+Na^+^).

#### 3.2.7. Procedure for the Preparation of 3-(4-Fluorobenzyl)-7-methoxy-2H-chromen-2-one (**84**) [25]

Step 1: Preparation of ethoxycarbonyl-(4-fluorobenzyl)-methylenetriphenylphosphorane (**8b**) [35]

To the solution of 4-fluorobenzyl bromide (0.378 g, 2 mmol) in dry chloroform (16 mL), ethoxycarbonylmethylene triphenylphosphorane (**7**, 0.68 g, 1.9 mmol) was added at room temperature. After stirring for 30 min at rt, it was refluxed for 15 hr. The solvent was removed under reduced pressure to give the phosphonium salt as an oil. This salt was dissolved in water (50 mL) and dichloromethane (50 mL) was added to it. It was then made alkaline (phenolphthalein colouless to pink) with 2 M NaOH. The dichloromethane layer was separated and the aqueous layer was extracted with dichloromethane (50 mL × 2). The combined dichloromethane extract was dried over MgSO_4_ and solvent was removed to give the phosphorane which was used without further purification for the next step.

Step 2: Preparation of (*E*)-ethyl 2-(4-fluorobenzyl)-3-(2-hydroxy-4-methoxyphenyl) acrylate (**9b**)

A mixture of **6** (0.189 g, 1.24 mmol) and **8b** (1.52 mmol) was refluxed at 85 °C in dry benzene (12 mL) for 15 h. The solvent was removed under reduced pressure. The residue obtained was purified by flash column chromatography (PE-EA = 5:1) to afford the crude product, which was recrystallised from chloroform-hexane as a white solid (0.206 g, 50%), m.p.: 114–115 °C. ^1^H-NMR (CDCl_3_): δ 1.24 (t, *J* = 7.2 Hz, 3H, CH_2_CH_3_), 3.79 (s, 3H, OCH_3_), 3.82 (s, 2H, CH_2_Ph), 4.20 (q, *J* = 7.2 Hz, 2H, CH_2_CH_3_), 5.10 (br s, 1H, OH), 6.44–6.47 (m, 2H, PhH), 6.92–6.97 (m, 2H, PhH), 7.00–7.13 (m, 3H, PhH), 7.90 (s, 1H, CH=C). MS (ESI): *m/z* 331.1 (M+H^+^), 348.1 (M+H_2_O), 353.1 (M+Na^+^), 369.1 (M+K^+^).

Step 3: Preparation of 3-(4-fluorobenzyl)-7-methoxy-2*H*-chromen-2-one (**84**)

Compound **9b** (0.66 g, 2 mmol) was heated at 220 °C for 3 h under a nitrogen atmosphere, The residue obtained was purified by flash column chromatography (PE-EA = 10:1) to afford the product, a white powder (0.408 g, 72%), m.p.: 94–95 °C. ^1^H-NMR (DMSO-*d*_6_): δ 3.76 (s, 2H, CH_2_Ph), 3.84 (s, 3H, OCH_3_), 6.92 (dd, *J* = 8.6, 2.4 Hz, 1H, H-6), 6.98 (d, *J* = 2.4 Hz, 1H, H-8), 7.11–7.16 (m, 2H, Ph-H), 7.33 (dd, *J* = 8.6, 5.4 Hz, 2H, Ph-H) 7.57 (d, *J* = 8.6 Hz, 1H, H-5), 7.75 (s, 1H, H-4). ^13^C-NMR (DMSO-*d*_6_): δ 35.46, 56.33, 100.85, 112.82, 113.13, 115.49, 115.70, 124.91, 129.45, 131.13, 135.21, 140.71, 154.92, 160.22, 161.41, 162.27, 162.62. HRMS (ESI): *m/z* 285.09185 (M+H^+^).

#### 3.2.8. Preparation of 3-Benzoyl-7-hydroxycoumarin (**85**) [26]

To a mixture of 2,4-dihydroxybenzylaldehyde (**10b**, 0.138 g, 1 mmol) and ethyl benzoylacetate (**16**, 0.192 g, 1.1 mmol) in acetonitrile (4 mL) was added 2 drops of piperidine. The neat mixture was kept stirring for 24 h at room temperature. The solvent was removed under reduced pressure. The products were purified by flash column chromatography (PE-EA = 3:1) to afford a yellow solid (0.23 g, 86.5%), m.p.: 220–221 °C. ^1^H-NMR (DMSO-*d*_6_): δ 6.81 (d, *J* = 2.0 Hz, 1H, H-8), 6.87 (dd, *J* = 8.8, 2.0 Hz, 1H, H-6), 7.53 (t, *J* = 7.6 Hz, 2H, *m*-Ph), 7.65–7.73 (m, 2H, *p*-Ph, H-5), 7.86 (d, *J* = 7.2 Hz, 2H, *o*-Ph), 8.35 (s, 1H, H-4), 11.05 (s, 0.11H, OH). MS (ESI): *m/z* 267.1 (M+H^+^), 289.0 (M+Na^+^), 305.0 (M+K^+^).

#### 3.2.9. Preparation of 3-benzoyl-7-methoxycoumarin (**86**) [26]

To a mixture of 2-hydroxyl-4-methoxybenzylaldehyde (**10a**, 0.152 g, 1 mmol, 1 equiv.) and ethyl benzoylacetate (**11**, 0.192 g, 1 mmol, 1 equiv.) was added piperidine (0.25 mL) dropwise. The neat mixture was kept stirring at room temperature until a pink solid was formed. The solid was then dissolved in CH_2_Cl_2_ and neutralised with 20 mL HCl (1 M). After extracting with CH_2_Cl_2_, the organic phase was dried over MgSO_4_ and concentrated. The crude product was then recrystallised from CH_2_Cl_2_–PE to give a pure product as yellow needle-like crystals (0.269 g, 96%), m.p.: 149–150 °C. ^1^H-NMR (CDCl_3_): δ 3.92 (s, 3H, OCH_3_), 6.87 (d, *J* = 2.0 Hz, 1H, H-8), 6.90–6.93 (dd, *J* = 8.8, 2.0 Hz, 1H, H-6), 7.46–7.51 (m, 3H, *m*-Ph, H-5), 7.58–7.60 (m, 1H, *p*-Ph), 7.85–7.87 (m, 2H, *o*-Ph), 8.10 (s,1H, H-4). ^13^C-NMR (CDCl_3_): δ 56.0, 100.7, 111.9, 113.6, 123.0, 128.5, 129.5, 130.5, 133.4, 136.8, 146.4, 157.2, 158.8, 164.7 (C=O), 192.0 (C=O). MS (ESI): *m/z* 281.1 (M+H^+^), 303.1 (M+Na^+^), 319.0 (M+K^+^).

#### 3.2.10. Preparation of3-Benzoyl-4-cyano-7-hydroxycoumarin (**87**) [27]

To the solution of **M19** (0.266 g, 1 mmol) in DMF (2 mL) was added sodium cyanide (0.098 g, 2 mmol), and the mixture was stirred at rt for 6 h. Then iodine was added to the solution and stirring at r.t. continued for 3 h. This solution was filtered and the residue washed with water and methanol and dried. The mixture was purified by flash column chromatography (PE-EA = 10:1) to afford the product as a yellow solid (0.097 g, 33.3%). M.p.: 193–194 °C. ^1^H-NMR (DMSO-*d*_6_): δ 6.90 (d, *J* = 2.4 Hz, 1H, H-8), 7.02 (dd, *J* = 8.8, 2.4 Hz, 1H, H-6), 7.58 (t, *J* = 7.6 Hz, 2H, *m*-Ph), 7.70–7.78 (m, 2H, *p*-Ph, H-5), 8.06 (d, *J* = 7.2 Hz, 2H, *o*-Ph), 11.33 (s, 1H, OH). HRMS (ESI): *m/z* 292.06037 (M+H^+^).

#### 3.2.11. Preparation of 3-Benzoyl-4-cyano-7-methoxycoumarin (**88**) [27]

To the solution of **86** (2.523 g, 9 mmol, 1 equiv.) in DMF (20 mL) was added sodium cyanide (0.864 g, 18 mmol, 2 equiv.), and the mixture was stirred at rt for 1 h. Then iodine (1.143 g, 9 mmol, 1 equiv.) was added to the solution and stirring continued at rt for 1 h. The mixture was processed by routine procedures and purified by flash column chromatography (PE-EA = 30:1) to afford the product as a yellow solid (0.76 g, 28%), m.p.: 175–176 °C. ^1^H-NMR (DMSO-*d*_6_): δ 3.93 (s, 3H, OCH_3_), 7.14–7.17 (dd, *J* = 8.8, 2.0 Hz, 1H, H-6), 7.2 (d, *J* = 2.0 Hz, 1H, H-8), 7.56–7.60 (t, *J* = 7.6 Hz, 2H, m-Ph), 7.74–7.77 (m, 2H, p-Ph, H-5), 8.08 (d, *J* = 7.2 Hz, 2H, o-Ph). ^13^C-NMR (DMSO-*d*_6_): δ 101.93, 109.79, 112.76, 114.53, 124.86, 128.32, 129.54, 130.26, 130.52, 135.42, 135.50, 156.12, 157.48, 165.15, 190.22. HRMS (ESI): *m/z* 306.07637 (M+H^+^), 328.05802 (M+Na^+^).

#### 3.2.12. Preparation of 3-Benzoyl-4-carboxy-7-methoxycoumarin (**89**) [28]

Compound **88** (100 mg, 0.33 mmol) in 50% H_2_SO_4_ (5 mL) was stirred at 100 °C for 15 h. The mixture was processed by routine procedures and purified by flash column chromatography (CH_2_Cl_2_–ethanol = 10:1) to afford the product as a yellow solid (0.03 g, 28%), m.p.: 196–197 °C. ^1^H-NMR (DMSO-*d*_6_): δ 3.92 (s, 3H, OCH_3_), 7.07 (dd, *J* = 8.8, 2.4 Hz, 1H, H-6), 7.15 (d, *J* = 2.4 Hz, 1H, H-8), 7.52 (t, *J* = 8.0 Hz, 2H, m-Ph), 7.65 (t, *J* = 7.6 Hz, 1H, p-Ph), 7.83 (d, *J* = 8.8 Hz, 1H, H-5), 7.90 (d, *J* = 8.0 Hz, 2H, o-Ph). ^13^C-NMR (DMSO-*d*_6_): δ 56.70, 101.58, 109.23, 113.83, 128.93, 129.15, 129.47, 134.02, 136.53, 156.49, 159.00, 164.00, 165.48. HRMS (ESI): *m/z* 325.07038 (M+H^+^).

### 3.3. MEK1 Binding Assay

The homogeneous time resolved fluorescence (HTRF) assay was performed on a Proxiplate-384 F plus solid back plate (Perkin Elmer, Waltham, MA. USA) with 5 nM GST tagged phosphorylated MEK1 (GST-MEK1), 2 nM europium-labeled anti-GST antibody (Eu-anti-GST), 20 nM kinase tracer 236 and test compound at a variety of concentrations. In the reaction mixture, GST-MEK1 forms a complex with the Eu-anti-GST and the tracer. Excitation of europium (the donor) using a 340-nm excitation filter results in energy transfer to the fluorophore of the tracer. This energy transfer is detected by an increase in the fluorescence emission of the tracer at 665 nm and a decrease in the fluorescence emission of europium at 615 nm. The FRET ratio was calculated by dividing the emission signal at 665 nm by the emission signal at 615 nm. A competitor compound such as a MEK inhibitor replaces the tracer from the complex and decreases the FRET ratio accordingly. The plate was incubated in dark at room temperature for 30 min before measuring the fluorescent emission of each well at 665 and 615 nm using a 340-nm excitation filter, 100-µs delay time, and 200-µs integration time, on a PHERAStar plate reader (BMG Labtech, Durham, NC, USA). AZD6244 (ARRY-142886) was used as positive control with 100.00% ± 1.45% inhibition at 100 µM. Data shown as %Inhibition normalized to AZD6244 at 100 µM. The curve-fitting software GraphPad Prism 4.0 was used to generate the curves and determine the IC_50_ values for individual compounds tested. %Inhibition = 100% × (DMSO-chemical)/(DMSO-AZD6244@100 μM).

### 3.4. Cell-Based Assays

#### 3.4.1. Cell Culture

Human Embryonic Kidney 293 cells (HEK 293) or rhabdomyosarcoma (RD) cells were grown in Dulbecco’s modified Eagle’s medium (DMEM, Thermo Scientific Hyclone, Logan, UT, USA) supplemented with 2% or 10% fetal bovine serum (FBS, GIBCO, Gaithersburg, MD, USA) at 37 °C in a humidified 5% CO_2_ incubator.

#### 3.4.2. AP-1 Luciferase Reporter Gene Assay

pAP1-Luc was constructed with pGL3-basic (Promega, Madison, WI. USA) and a fragment including the AP-1 response element (2× TRE) and T7 promoter. HEK 293 cells in 96-well plates were cultured at 5% CO_2_ at 37 °C in DMEM containing 10% FBS. When the confluence was up to 80%, cells were transinfected with pAP1-Luc using lipofectamine 2000 (Life Technologies, Carlsbad, CA. USA). At 24 h post transinfection, individual compounds with different concentrations (5, 10, 20, 30 μM) were added into cells using three wells for each concentration used. After incubation for 1 h, TPA (50 nM) was added. The cells were harvested after 4 h and luciferase activity was measured by using luciferase assay kit (Promega) with a luminometer. The concentration of 50% inhibition (IC_50_) of each compound was calculated.

#### 3.4.3. Antiviral Assay

RD cells were cultured in DMEM and up to 70%–80% confluence in 96-well plates. Compounds were added into the culture medium for 1 h prior to virus infection using four wells for each concentration used. The compounds were added at the following concentrations: 1.25, 2.5, 5, 10, 20, 40 μM. Then cells were infected withEV71 (a clinical strain denoted as EV71-BC08, GU475127) at a multiplicity of infection (MOI) of 1. Virus controls (untreated wells of infected cells), cell controls (cells and drugs only, using the same dilution range for each compound as the test wells) were included on the plate. The cytopathic effect (CPE) were examined at every 12 h intervals post-infection by using phase-contrast microscopy. At 48 h after infection, the virus control wells showed 100% cytopathology. The 50% effective concentration (EC_50_) was determined for each compound through Reed-Muench method. The cytotoxicity (CC_50_) was assayed by the MTT method.

## 4. Conclusions

Compounds **13**, **18**, **19** and **33** have shown good activity in all the above assays. The common sub-structure they share is *N, N*-dimethylcarbamoyloxyl or acetoxyl substitution at the C7 position, and this implies the importance of substituents at this position. Besides the hydrogen bond formed by the carbonyl group of the *N, N*-dimethylcarbamoyloxyl or acetoxyl groups and Asp208, the activity may be related to the steric hindrances and specific carbon chain lengths of these substituent groups. 

In summary, using a computer-aided drug design (CADD) method, we have developed a series of novel 3-substituted benzylcoumarins as allosteric MEK1 inhibitors. Some of the compounds showed excellent MEK1 binding affinity and displayed obvious inhibitory effects on the ERK pathway. Functionally, they could significantly inhibit replication of viruses such as EV71 in a cell-based assay. These compounds may overcome the drug resistance problem caused by virus variation and might be potential candidates for the treatment of viral diseases. Further structure optimizations and investigations to improve the potency and explore the pharmacological properties of these new MEK1 inhibitors are ongoing in our group. 

## Abbreviations

MAPK: Mitogen-activated protein kinase
MAP3K: Mitogen-activated protein kinase kinase kinase
MEK: mitogen-activated protein kinase/extracellular signal regulated kinase kinase
ERK: mitogen-activated protein kinase/extracellular signal-regulated kinase
EV71: Enterovirus 71
HEK293: human embryonic kidney293
RD cell: rhabdomyosarcoma cell
PDB: protein data bank
HTRF: homogeneous time-resolved fluorescence
FRET: Förster (Fluorescence) resonance energy transfer
TPA: 12-*O*-tetradecanoylphorbol-13-acetate
AP-1: activating protein-1
PE: petroleum ether
EA: ethyl acetate
Ts: tosyl
*t*-Bu: *tert*-butyl

## Supplementary Materials

Results for the *in vitro* biological studies of all compounds. Supplementary materials can be accessed at: http://www.mdpi.com/1420-3049/18/5/6057/s1.

## References

[1] Johnson R.A., Ma X.L., Yurochko A.D., Huang E.S. (2001). The role of MKK1/2 kinase activity in human cytomegalovirus infection. J. Gen. Virol..

[2] Ludwig S., Wolff T., Ehrhardt C., Wurzer W.J., Reinhardt J., Planz O., Pleschka S. (2004). MEK inhibition impairs influenza B virus propagation without emergence of resistant variants. FEBS Lett..

[3] Yi T., Zhang H., Peng Y.-H., Zhu M., He X.-Y., Huang X.-T. (2011). Activation of MEK1-ERKs is essential for herpes simplex virus type 2 (HSV2) replication. Zhongguo Shengwu Huaxue Yu Fenzi Shengwu Xuebao.

[4] Pei R., Zhang X., Xu S., Meng Z., Roggendorf M., Lu M., Chen X. (2012). Regulation of Hepatitis C virus replication and gene expression by the MAPK-ERK pathway. Virol. Sin..

[5] Andrade A.A., Silva P.N.G., Pereira A.C.T.C., de Sousa L.P., Ferreira P.C.P., Gazzinelli R.T., Kroon E.G., Ropert C., Bonjardim C.A. (2004). The vaccinia virus-stimulated mitogen-activated protein kinase (MAPK) pathway is required for virus multiplication. Biochem. J..

[6] Price S. (2008). Putative allosteric MEK1 and MEK2 inhibitors. Expert Opin. Ther. Pat..

[7] Fremin C., Meloche S.  (2010). From basic research to clinical development of MEK1/2 inhibitors for cancer therapy. J. Hematol. Oncol..

[8] Ohren J.F., Chen H.F., Pavlovsky A., Whitehead C., Zhang E.L., Kuffa P., Yan C.H., McConnell P., Spessard C., Banotai C. (2004). Structures of human MAP kinase kinase 1 (MEK1) and MEK2 describe novel noncompetitive kinase inhibition. Nat. Struct. Mol. Biol..

[9] Cozza G., Bortolato A., Menta E., Cavalletti E., Spinelli S., Moro S. (2009). ATP non-competitive Ser/Thr kinase inhibitors as potential anticancer agents. Anticancer Agents Med. Chem..

[10] Wang D., Boerner S.A., Winkler J.D., LoRusso P.M. (2007). Clinical experience of MEK inhibitors in cancer therapy. Biochim. Biophys. Acta.

[11] Kiselyov A., Balakin K.V., Tkachenko S.E., Savchuk N.P. (2006). Recent progress in development of non-ATP competitive small-molecule inhibitors of protein kinases. Mini Rev. Med. Chem..

[12] Harrison S., Das K., Karim F., Maclean D., Mendel D. (2008). Non-ATP-competitive kinase inhibitors-enhancing selectivity through new inhibition strategies. Expert Opin. Drug Discov..

[13] Trujillo J.I. (2011). MEK inhibitors: A patent review 2008–2010. Expert Opin. Ther. Pat..

[14] Heald R.A., Jackson P., Savy P., Jones M., Gancia E., Burton B., Newman R., Boggs J., Chan E., Chan J. (2012). Discovery of novel allosteric mitogen-activated protein kinase kinase (MEK) 1,2 inhibitors possessing bidentate Ser212 interactions. J. Med. Chem..

[15] Wang J., Wilcoxen K.M., Nomoto K., Wu S. (2007). Recent advances of MEK inhibitors and their clinical progress. Curr. Top. Med. Chem..

[16] Dudley D.T., Pang L., Decker S.J., Bridges A.J., Saltiel A.R. (1995). A synthetic inhibitor of the mitogen-activated protein kinase cascade. Proc. Natl. Acad. Sci. USA.

[17] Wang B., Ding L., Deng J., Zhang H., Zhu M., Yi T., Liu J., Xu P., Lu F., Peng Y. (2010). Replication of EV71 was suppressed by MEK1/2 inhibitor U0126. Chin. J. Biochem. Mol. Biol..

[18] Tu J., Zhang H., Zhou Q., Wang B., Cao M., Huang H., Peng Y. (2008). Key role of MEK1/2-ERK pathway in replication of type II herpes simplex virus (HSV-2). Chin. J. Biochem. Mol. Biol..

[19] Luo L., Tu J., Feng H., Zhang H., Peng Y. (2007). Effects of the host cell ERK signal transduction pathway on replication of type II herpes simplex virus (HSV-2). Chin. J. Biochem. Mol. Biol..

[20] Peng Y. (2012). ERK signal cascade: The host cell’s mechinery for viral reproduction and its implication as the potential anti-viral target. Chin. J. Viral Dis..

[21] He J., Duan H., Peng Y. (2012). ERK/MAPK pathway and replication, pathogenesis of human RNA virus:a short review. Chin. J. Viral Dis..

[22] Wang B., Zhang H., Zhu M., Luo Z., Peng Y. (2012). MEK1-ERKs signal cascade is required for the replication of Enterovirus 71 (EV71). Antiviral Res..

[23] Han S., Zhou V., Pan S., Liu Y., Hornsby M., McMullan D., Klock H.E., Haugen J., Lesley S.A., Gray N. (2005). Identification of coumarin derivatives as a novel class of allosteric MEK1 inhibitors. Bioorg. Med. Chem. Lett..

[24] Cheng J.F., Arrhenius T., Wallace D., Chen M., Tith S., Kashiwagi H., Ono Y., Watanabe Y. (2002). Preparation of Coumarin Derivatives Useful as TNFα Inhibitors. W.O. Patent.

[25] Britto N., Gore V.G., Mali R., Ranade A. (1989). A convenient synthesis of 3-behzyl, 3-benzyl-4-substituted coumarins and their benzo derivatives. Synth. Commun..

[26] Tang X., Blake A.J., Lewis W., Woodward S. (2009). Asymmetric conjugate additions to 1,1′-diactivated cyclic enones—A comparative study. Tetrahedron Asymmetry.

[27] Lee S., Sivakumar K., Shin W.-S., Xie F., Wang Q. (2006). Synthesis and anti-angiogenesis activity of coumarin derivatives. Bioorg. Med. Chem. Lett..

[28] Brubaker A.N., de Ruiter J., Whitmer W.L. (1986). Synthesis and rat lens aldose reductase inhibitory activity of some benzopyran-2-ones. J. Med. Chem..

[29] Muller K., Faeh C., Diederich F. (2007). Fluorine in pharmaceuticals: Looking beyond intuition. Science.

[30] Purser S., Moore P.R., Swallow S., Gouverneur V. (2008). Fluorine in medicinal chemistry. Chem. Soc. Rev..

[31] Hagmann W.K. (2008). The many roles for fluorine in medicinal chemistry. J. Med. Chem..

[32] Ameyar M., Wisniewska M., Weitzman J.B. (2003). A role for AP-1 in apoptosis: The case for and against. Biochimie.

[33] Karin M., Liu Z.G., Zandi E. (1997). AP-1 function and regulation. Curr. Opin. Cell Biol..

[34] Favata M.F., Horiuchi K.Y., Manos E.J., Daulerio A.J., Stradley D.A., Feeser W.S., van Dyk D.E., Pitts W.J., Earl R.A., Hobbs F. (1998). Identification of a novel inhibitor of mitogen-activated protein kinase kinase. J. Biol. Chem..

[35] Mali R.S., Yadav V.J., Zaware R.N.  (1982). An improved synthesis of naturally occurring coumarins: Synthesis of herniarin, scoparone & 7-methoxy-6-methylcoumarin. Indian J. Chem. Sect. B: Org. Chem. Incl. Med. Chem..

