# Supplementary material for: Substituted 3-Benzylcoumarins as Allosteric MEK1 Inhibitors: Design, Synthesis and Biological Evaluation as Antiviral Agents
Source: Molecules. 2013 May 21;18(5):6057–91. doi: 10.3390/molecules18056057 (PMC6269873; doi:10.3390/molecules18056057)

Supplementary Materials

Biological Assays

Figure S1. Binding affinity of AZD6244 and compounds **13–18**.

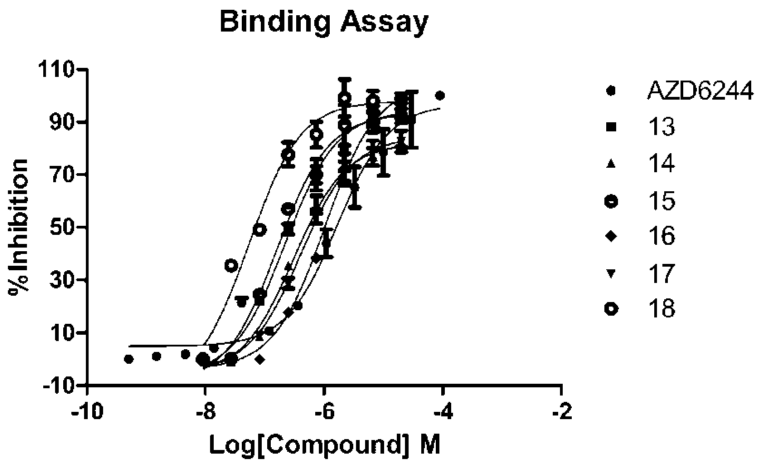

Figure S2. Binding affinity of compounds **19**, **22**, **25**, **28**, **29**, **31**.

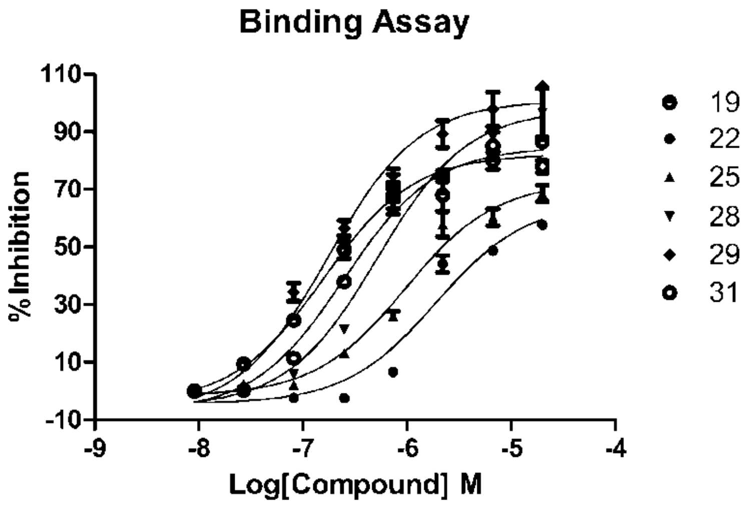

Figure S3. Binding affinity of compounds **32–36**, **38–39**.

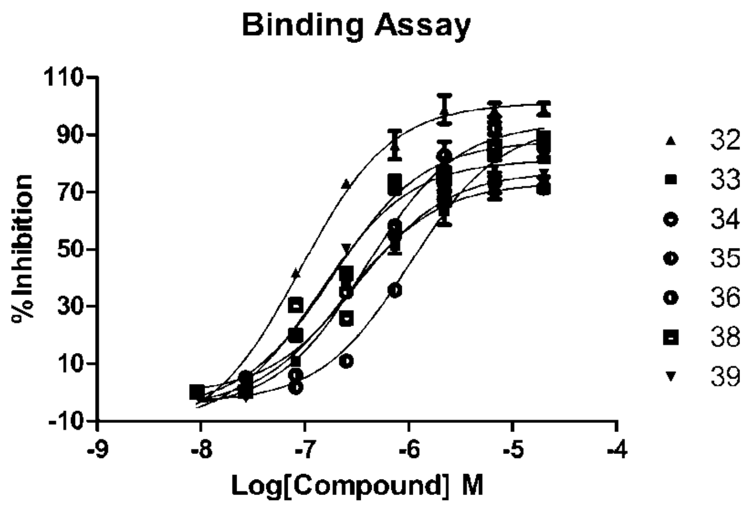

Figure S4. Binding affinity of compounds **40**, **42**, **43**, **45**–**47**.

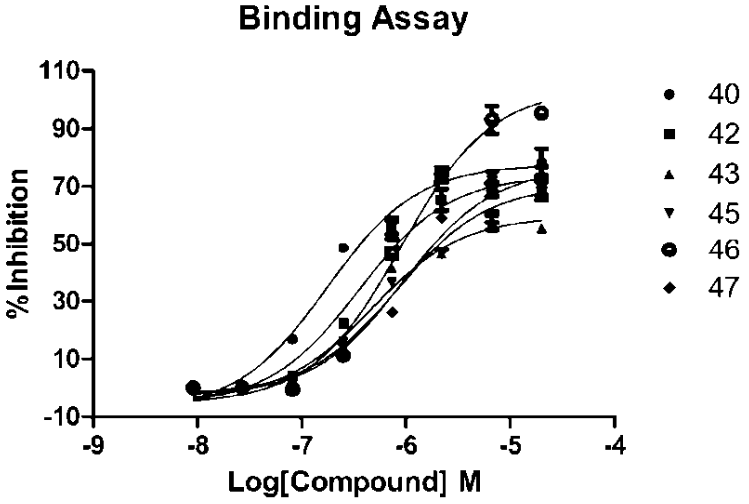

Supplement: Supplementary file 1 [file molecules-18-06057-s001.pdf]
